# Patient-specific closed-loop model of the fontan circulation: Calibration and validation

**DOI:** 10.1016/j.heliyon.2024.e30404

**Published:** 2024-04-26

**Authors:** Jorge Aramburu, Bram Ruijsink, Radomir Chabiniok, Kuberan Pushparajah, Jordi Alastruey

**Affiliations:** a Universidad de Navarra, TECNUN Escuela de Ingeniería, P° Manuel Lardizabal 13, 20018, Donostia/San Sebastián, Spain; b School of Biomedical Engineering and Imaging Sciences, King's College London, St Thomas' Hospital, SE1 7EH, London, UK; c Division of Pediatric Cardiology, Department of Pediatrics, UT Southwestern Medical Center, Dallas, TX, USA; d Department of Congenital Heart Disease, Evelina Children's Hospital, SE1 7EH, London, UK

**Keywords:** Fontan circulation, 1-D model, Congenital heart disease, Parameter estimation

## Abstract

The Fontan circulation, designed for managing patients with a single functional ventricle, presents challenges in long-term outcomes. Computational methods offer potential solutions, yet their application in cardiology practice remains largely unexplored. Our aim was to assess the ability of a patient-specific, closed-loop, reduced-order blood flow model to simulate pulsatile blood flow in the Fontan circulation. Using one-dimensional models, we simulated the aorta, superior and inferior venae cavae, and right and left pulmonary arteries, while lumping heart chambers and remaining vessels into zero-dimensional models. The model was calibrated with patient-specific haemodynamic data from combined cardiac catheterisation and magnetic resonance exams, using a novel physics-based stepwise methodology involving simpler open-loop models. Testing on a 10-year-old, anesthetised patient, demonstrated the model's capability to replicate pulsatile pressure and flow in the larger vessels and ventricular pressure. Average relative errors in mean pressure and flow were 2.9 % and 3.6 %, with average relative point-to-point errors (RPPE) in pressure and flow at 5.2 % and 16.0 %. Comparing simulation results to measurements, mean aortic pressure and flow values were 50.7 vs. 50.4 mmHg and 41.6 vs. 41.9 ml/s, respectively, while ventricular pressure values were 28.7 vs. 27.4 mmHg. The model accurately described time-varying ventricular volume with a RPPE of 2.9 %, with mean, minimum, and maximum ventricular volume values for simulation results vs. measurements at 59.2 vs. 58.2 ml, 38.0 vs. 37.6 ml, and 76.0 vs. 74.4 ml, respectively. It provided physiologically realistic predictions of haemodynamic changes from pulmonary vasodilation and atrial fenestration opening. The new model and calibration methodology are freely available, offering a platform to virtually investigate the Fontan circulation's response to clinical interventions and explore potential mechanisms of Fontan failure. Future efforts will concentrate on broadening the model's applicability to a wider range of patient populations and clinical scenarios, as well as testing its effectiveness.

## Abbreviations

0-D: zero-dimensional
1-D: one-dimensional
3-D: three-dimensional
3WK: three-element Windkessel
AAo: ascending aorta
AoV: aortic valve
AVV: atrioventricular valve
BCA: brachiocephalic artery
CMR: cardiovascular magnetic resonance
CO: cardiac output
DAo: descending aorta
HR: heart rate
iCMR: invasive cardiovascular magnetic resonance (combined cardiac catheterisation and cardiovascular magnetic resonance imaging)
IVC: inferior vena cava
LCCA: left common carotid artery
LPA: left pulmonary artery
LPCW: left pulmonary capillary wedge
LSA: left subclavian artery
MRI: magnetic resonance imaging
PVR: pulmonary vascular resistance
PWV: pulse wave velocity
RPA: right pulmonary artery
RPPE: relative point-to-point error
SVC: superior vena cava
SV: stroke volume
SVR: systemic vascular resistance
TCPC: total cavopulmonary connection

## 1 Introduction

A functionally univentricular heart is a rare congenital heart condition that affects 4 to 8 out of every 10,000 newborns [1]. It includes conditions such as hypoplastic left or right heart syndrome (i.e. underdeveloped left or right heart ventricle, respectively) due to conditions such as mitral or tricuspid valve atresia. Typically, it leads to hypoxemia (i.e. low levels of oxygen in the blood) caused by the mixing of venous returns from the systemic and pulmonary circulations [2]. Patients undergo a three-stage procedure to help restore heart function, culminating in the Fontan operation [2], pioneered by Fontan and Baudet in the 1970s [3]. This operation connects the superior vena cava (SVC) and the inferior vena cava (IVC) directly to the right pulmonary artery (RPA) and left pulmonary artery (LPA) via the total cavopulmonary connection (TCPC), improving oxygenation and prolonging patient survival [4]. In this surgically created circulation, only well-oxygenated blood reaches the heart since the systemic venous return is directly connected to the pulmonary arteries. However, this setup requires the heart to do about 40 % more work than a normal heart, as only one ventricle pumps blood for the entire circulation—systemic and pulmonary circulations connected in series [5].

Recent advances in surgery and perioperative care have improved Fontan patient survival [4]. However, long-term outcomes remain challenging, which calls for the integration of novel methods such as computational modelling into cardiology practice [[6], [7], [8]]. The Fontan circulation has been simulated using three-dimensional (3-D), one-dimensional (1-D), and zero-dimensional (0-D) haemodynamic models. Studies using 3-D models have primarily aimed to quantify the efficiency of blood flow in the TCPC, focusing on power loss and blood flow distribution [[9], [10], [11], [12]]. For example, Ding et al. found that hepatic flow to lungs and the RPA to LPA flow ratio are sensitive to the angle between the IVC and RPA, while power loss is sensitive to the IVC-SVC angle [13]. Yang et al. compared the performance of T-shaped, Y-shaped, and offset designs by virtually implanting the models in five patient-specific Glenn models. Their findings revealed that the Y-shaped design improved hepatic flow distribution and offered improvements in power loss and SVC pressure [14]. Subsequently, Yang et al. evaluated the postoperative haemodynamic performance of patients undergoing handcrafted Y-graft Fontan procedure and validated simulation predictions using *in vivo* clinical data [15]. Rajabzadeh-Oghaz et al. compared power loss and flow pulsatility in T-shaped and Y-shaped grafts, concluding that Y-shaped geometries produced much lower power losses than T-shaped geometries, with no difference in flow pulsatility [16]. Lastly, Siallagan et al. designed patient-specific 3-D models of Fontan geometry, aiming to enhance hepatic flow distribution, reduce energy loss, and manufacture these designs by electrospinning [17].

On the other hand, 1-D models have been used to simulate flow and pressure pulse wave propagation throughout the entire Fontan circulation and investigate the impact of surgical interventions. For instance, Puelz et al. analysed modifications to extracardiac Fontan haemodynamics, finding that both fenestration and hepatic vein exclusion increased intestinal flow, with hepatic vein exclusion notably reducing portal venous pressure and fenestration decreasing pulmonary artery pressure [18]. Similarly, 0-D models have been widely used to simulate the entire Fontan circulation and study the influence of surgical interventions [19]. Watrous and Chin described healthy and Fontan circulations using a 0-D model [20]. Di Molfeta et al. calibrated 0-D models with data from four pigs for normal and Fontan circulations, including ventricular assist device scenarios [21]. Hsu et al. studied the effects of a cavopulmonary assist device and veno-arterial extracorporeal membrane oxygenation on failing Fontan [22]. Conover et al. created a web-based tool using a 0-D single-ventricle model for quick, accurate, patient-specific haemodynamic predictions to aid decision-making, training, and consultation [23]. Additionally, 0-D models have shown great potential for assessing combined ventricle-circulation function [24]. In the adult circulation, reduced-order 1-D and 0-D models can simulate pulse wave propagation in large arteries with reasonable computational cost and comparable accuracy to 3-D models [25]. However, no assessment of 1-D/0-D modelling accuracy in the Fontan circulation against *in vivo* data has been conducted yet.

In this study, we aimed to assess the ability of reduced-order modelling to simulate blood flow in the entire Fontan circulation of a 10-year-old. We created a patient-specific, closed-loop 1-D/0-D model, with larger vessels described using the 1-D formulation and the remaining circulation using 0-D models. This approach enables geometrical variations, such as aortic coarctation or compression of the LPA by the aorta. Additionally, we developed a novel physics-based method for model calibration using patient-specific data from combined cardiac catheterisation and cardiac magnetic resonance (CMR) exams; i.e. an invasive CMR (iCMR) [26] exam. Our model showed promising results in two clinically realistic scenarios: administration of a pulmonary vasodilator and opening of an atrial fenestration. The novel model and calibration method are freely available at https://zenodo.org/records/11076659 to support further research on Fontan circulation haemodynamics.

## 2 Methods

### 2.1 Patient data

**Fig. 1:**
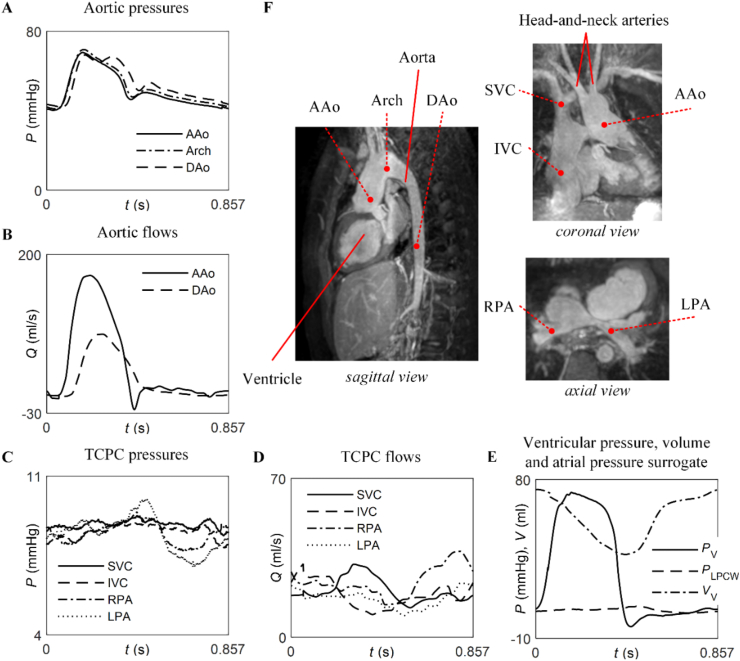
Haemodynamic data available to calibrate the model. *In vivo* pressure, flow, and volume measurements with time (***A***–***E***), together with images acquired during the iCMR exam (***F***). Red circles indicate the locations where the measurements were taken. AAo: Ascending aorta; Arch: Transverse arch; DAo: descending aorta; IVC: inferior vena cava; LPA: left pulmonary artery; LPCW: left pulmonary capillary wedge; RPA: right pulmonary artery; SVC: superior vena cava. (For interpretation of the references to colour in this figure legend, the reader is referred to the Web version of this article.)

Haemodynamic data were obtained from an iCMR exam (Philips Achieva 1.5 T MRI system) in a 10-year-old, anesthetised patient with hypoplastic left heart syndrome and Fontan circulation after staged palliation surgery, who experienced progressive exercise intolerance (early-stage heart failure). Table 1 and Fig. 1 show these data. Invasive pressure measurements were taken in the ascending aorta (AAo), aortic arch and descending aorta (DAo) (Fig. 1A); SVC, IVC, LPA and RPA (Fig. 1C); and ventricle (Fig. 1E). A left pulmonary capillary wedge (LPCW) pressure was obtained to assess the transpulmonary pressure gradient (Fig. 1E). The flow at all pressure measurements sites, except the ventricle and aortic arch, was measured by two-dimensional, phase-contrast magnetic resonance imaging (MRI) (Fig. 1B and D). In the ventricle, blood pool volume was measured from cine MRI (Fig. 1E). The calibre and length of the aortic branches and TCPC connections were manually measured, directly from the magnetic resonance images (Fig. 1F). During the scan, it was noted that the origin of the left subclavian artery (LSA) was not clearly visible, and the distal vessel of the LSA was filled late, possibly due to retrograde filling through collaterals from the remaining head and neck vasculature. No aorto-pulmonary or veno-venous collaterals were detected in this patient, and there was no patent fenestration. This study was conducted under the ethical approval of our institutional ethics committee in London, UK (Ethics Number 09H0804062), with written consent.

**Table 1:** Haemodynamic data acquired from the iCMR exam.

Heart rate (bpm)	70
*Ventricular volumes*	
End-diastolic volume (ml)	74.4
End-systolic volume (ml)	37.6
Stroke volume (ml)	36.8
Cardiac output (l/min)	2.51
Ejection fraction (%)	48
*CMR flow measurements*	
AAo (ml/beat)	36
DAo (ml/beat)	16
RPA (ml/beat)	21
LPA (ml/beat)	14
SVC (ml/beat)	18
IVC (ml/beat)	17
*Invasive pressures*	
Ventricular end-diastolic pressure (mmHg)	7
Ventricular peak pressure (mmHg)	73
AAo, systolic/diastolic (mean) (mmHg)	69/40 (50)
Transverse arch, systolic/diastolic (mean) (mmHg)	71/41 (52)
DAo, systolic/diastolic (mean) (mmHg)	68/42 (53)
IVC, mean (mmHg)	8.5
SVC, mean (mmHg)	8.9
LPA, mean (mmHg)	8.4
RPA, mean (mmHg)	8.4
LPCW, mean (mmHg)	6
Measured PVR (mmHg·min/l)	1.2

AAo: ascending aorta; DAo: descending aorta; IVC: inferior vena cava; LPA: left pulmonary artery; LPCW: left pulmonary capillary wedge; PVR: pulmonary vascular resistance; SVC: superior vena cava; RPA: right pulmonary artery.

### 2.2 Fontan circulation model

**Fig. 2:**
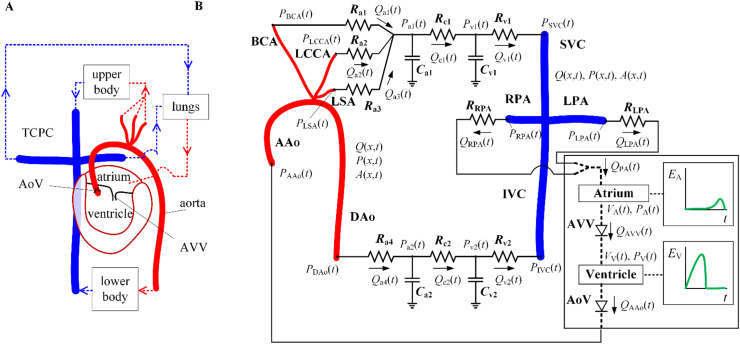
Schematics of the Fontan circulation 1-D/0-D model. ***A***: The main components of the model are the aorta, total cavopulmonary connection (TCPC), and the lumped upper body, lower body, lungs, and heart. ***B***: The 1-D model aorta is coupled to the TCPC via 0-D models of the upper and lower bodies, lungs and heart. The aorta consists of the ascending aorta (AAo), the brachiocephalic artery (BCA), the left common carotid artery (LCCA), the left subclavian artery (LSA), and the descending aorta (DAo). The TCPC consists of the superior vena cava (SVC), the inferior vena cava (IVC), the right pulmonary artery (RPA), and the left pulmonary artery (LPA). The heart consists of the atrium, the atrioventricular valve (AVV), the ventricle, and the aortic valve (AoV). Blood flow is produced by the action of the time-varying elastance of ventricle (*E*_V_) and atrium (*E*_A_). All model parameters are described in Table 2. Arch: aortic arch; LPCW: left pulmonary capillary wedge.

Fig. 2 depicts a schematic representation of the closed-loop, 1-D/0-D model of the Fontan circulation. Our goal with this model was to minimise the total number of input parameters and maximise the number of patient-specific input parameters, while preserving reasonable accuracy in the computed haemodynamic signals. The aorta and TCPC were modelled using the 1-D formulation, while the remaining parts of the cardiovascular system—upper body, lower body, lungs, and heart—were lumped into 0-D Windkessel models [25]. Two time-varying elastance functions were prescribed to simulate the filling and contraction of the atrium and the ventricle. The following sections describe the model's governing equations and the methodology used to estimate their parameters. In addition to the parameters presented in these sections, the cardiac cycle period (*T*) and total blood volume (*V*_blood_) were also prescribed. Table 2 and Table 3 show all model parameters.

**Table 2:** Model parameters, their values in the patient-specific case of this study, and their descriptions. The parameters with an asterisk (*) were manually adjusted and the remaining parameters were estimated from the patient's data.

PARAMETERS	VALUE	DESCRIPTION
HEART MODEL PARAMETERS		
*Atrium parameters*		
*K*_s,A_ (s/ml)*	7.5·10^−4^	Source resistance coefficient for the atrium
*E*_min,A_ (mmHg/ml)*	0.2	Minimum atrial elastance
*E*_max,A_ (mmHg/ml)*	0.4	Maximum atrial elastance
*V*_0,A_ (ml)*	5.5	Reference volume for the atrium
*m*_1,A_ (−)*	20	Atrial contraction rate constant
*m*_2,A_ (−)*	30	Atrial relaxation rate constant
*τ*_1,A_/*T* (−)*	0.1	Atrial systolic time constant per unit cardiac cycle period
*τ*_2,A_/*T* (−)*	0.25	Atrial diastolic time constant per unit cardiac cycle period
*t*_onset,A_/*T* (−)*	0.7	Onset of atrial contraction per unit cardiac cycle period
*Atrioventricular valve parameters*		
*L*_eff,AVV_ (cm)*	4	Effective length of atrioventricular valve (AVV)
*A*_ann,AVV_ (cm^2^)*	4.5	Annulus area of AVV
*K*_vo,AVV_ (mmHg^−1^·s^−1^)*	8	AVV opening rate coefficient
*K*_vc,AVV_ (mmHg^−1^·s^−1^)*	8	AVV closing rate coefficient
*Ventricle parameters*		
*K*_s,V_ (s/ml)	1.4·10^−4^	Source resistance coefficient for the ventricle
*E*_min,V_ (mmHg/ml)*	0.065	Minimum ventricular elastance
*E*_max,V_ (mmHg/ml)*	1.97	Maximum ventricular elastance
*V*_0,V_ (ml)*	10.5	Reference volume for the ventricle
*m*_1,V_ (−)*	1.3	Ventricular contraction rate constant
*m*_2,V_ (−)*	30	Ventricular relaxation rate constant
*τ*_1,V_/*T* (−)*	0.18	Ventricular systolic time constant per unit cardiac cycle period
*τ*_2,V_/*T* (−)*	0.45	Ventricular diastolic time constant per unit cardiac cycle period
*t*_onset,V_/*T* (−)*	0	Onset of ventricular contraction per unit cardiac cycle period
*Aortic valve parameters*		
*L*_eff,AoV_ (cm)*	8	Effective length of aortic valve (AoV)
*A*_ann,AoV_ (cm^2^)*	1.68	Annulus area of AoV
*K*_vo,AoV_ (mmHg^−1^·s^−1^)*	4	AoV opening rate coefficient
*K*_vc,AoV_ (mmHg^−1^·s^−1^)*	2	AoV closing rate coefficient
UPPER BODY, LOWER BODY, AND LUNG MODEL PARAMETERS		
*Upper body*		
*R*_a1_ (mmHg·s/ml)	0.5086	Arterial resistance to blood flow connected to BCA
*R*_a2_ (mmHg·s/ml)	2.2430	Arterial resistance to blood flow connected to LCCA
*R*_a3_ (mmHg·s/ml)	–	Arterial resistance to blood flow connected to LSA
*R*_c1_ (mmHg·s/ml)	1.3305	Capillary resistance to blood flow in the upper body
*R*_v1_ (mmHg·s/ml)	0.0054	Venous resistance to blood flow in the upper body
*C*_a1_ (ml/mmHg)	2.4563	Arterial compliance in the upper body
*C*_v1_ (ml/mmHg)	23.3356	Venous compliance in the upper body
*Lower body*		
*R*_a4_ (mmHg·s/ml)	0.2646	Arterial resistance to blood flow connected to DAo
*R*_c2_ (mmHg·s/ml)	0.5767	Capillary resistance to blood flow in the lower body
*R*_v2_ (mmHg·s/ml)	1.4841	Venous resistance to blood flow in the lower body
*C*_a2_ (ml/mmHg)	2.4921	Arterial compliance in the lower body
*C*_v2_ (ml/mmHg)	0.1897	Venous compliance in the lower body
*Lungs*		
*R*_RPA_ (mmHg·s/ml)	0.1720	Resistance to blood flow of RPA
*R*_LPA_ (mmHg·s/ml)	0.2474	Resistance to blood flow of LPA
ADDITIONAL MODEL PARAMETERS		
*T* (s)	0.857	Cardiac cycle period
*V*_blood_ (ml)	556.69	Total blood volume in the model
*c*_Ao_ (m/s)	5.35	PWV in the aorta, BCA, and LCCA
*c*_TCPC_ (m/s)	2.81	PWV in the TCPC

**Table 3:** Characteristics of the one-dimensional model vessels. Domain and node nomenclature, length (*L*), inlet diameter (*D*_in_), outlet diameter (*D*_out_), and pulse wave velocity (PWV). Domain and node numbering can be seen in Fig. S4 in online-only Data Supplement.

Vessel	Domain	Node (in)	Node (out)	*L* (mm)	*D*_in_ (mm)	*D*_out_ (mm)	PWV (m/s)
AAo	1	1	2	29.0	25.0	15.5	5.35
DAo	2	2	3	90.0	15.5	10.5	5.35
BCA	3	2	4	25.0	9.0	10.5	5.35
LCCA	4	2	5	30.0	6.5	5.0	5.35
IVC	5	6	7	55.0	31.0	22.0	2.81
RPA	6	7	10	12.0	13.0	13.0	2.81
LPA (i)	7	7	8	22.0	10.5	6.0	2.81
LPA (ii)	8	8	11	14.0	6.0	8.0	2.81
SVC	9	7	9	15.0	22.0	14.5	2.81

#### 2.2.1 Univentricular heart

Blood flow in the univentricular heart was simulated based on the model by Mynard et al. [27], consisting of the following elements connected in series (see Fig. 2B): (i) the atrium defined by its volume (*V*_A_(*t*), where *t* is time) and pressure (*P*_A_(*t*)), with an input flow from the pulmonary arteries (*Q*_PA_(*t*)) and an output flow towards the atrioventricular valve (AVV) (*Q*_AVV_(*t*)); (ii) the AVV; (iii) the ventricle defined by its volume (*V*_V_(*t*)) and pressure (*P*_V_(*t*)), with an input flow from the AVV (*Q*_AVV_(*t*)) and an output flow towards the aortic valve (AoV) and therefore the ascending aorta (*Q*_AAo_(*t*)); and (iv) the AoV.

Blood in the atrium is governed by a conservation of mass equation (Eq. (1)) and the atrial pressure is produced by a time-varying elastance (*E*_A_(*t*)), (Eq. (2)),

(1) $$\frac{dV_{A}}{dt}=Q_{\text{PA}}-Q_{\text{AVV}}$$

(2) $$P_{A}=E_{A}\left(V_{A}-V_{0,A}\right)\left(1-K_{s,A}Q_{\text{AVV}}\right)$$

where *V*_0,A_ is a reference volume for the atrium and *K*_s,A_ is a source resistance coefficient. *E*_A_(*t*) is described by Eqs. (3), (4), (5),

(3) $$E_{A}=k_{A}\frac{g_{1,A}}{1+g_{1,A}}\frac{1}{1+g_{2,A}}+E_{\min,A}$$

(4) $$g_{1,A}={\left(\frac{t-t_{\text{onset},A}}{\tau_{1,A}}\right)}^{m_{1,A}},g_{2,A}={\left(\frac{t-t_{\text{onset},A}}{\tau_{2,A}}\right)}^{m_{2,A}}$$

(5) $$k_{A}=\frac{E_{\max,A}-E_{\min,A}}{\max\left(\frac{g_{1,A}}{1+g_{1,A}}\frac{1}{1+g_{2,A}}\right)}$$

where *E*_max,A_ and *E*_min,A_ are the maximum and minimum elastance values of the atrium, respectively, *τ*_1,A_ and *τ*_2,A_ are the systolic and diastolic time constants, respectively, *m*_1,A_ and *m*_2,A_ are the contraction and relaxation rate constants, respectively, *k*_A_ is a scaling factor to ensure that the maximum value of *E*_A_ equals *E*_max,A_, and *t*_onset,A_ is the onset time for atrial contraction.

Blood flow across the AVV is governed by Bernoulli's equation as described in Eqs. (6), (7), (8), which relate the pressure difference and the flow across an open valve,

(6) $$\frac{dQ_{\text{AVV}}}{dt}=\frac{1}{L_{\text{AVV}}}\left(P_{A}-P_{V}-B_{\text{AVV}}\left|Q_{\text{AVV}}\right|Q_{\text{AVV}}\right)$$

(7) $$B_{\text{AVV}}=\frac{\rho }{2A_{\text{eff},\text{AVV}}^{2}},\;L_{\text{AVV}}=\frac{\rho l_{\text{eff},\text{AVV}}}{A_{\text{eff},\text{AVV}}},\;A_{\text{eff},\text{AVV}}=A_{\text{ann},\text{AVV}}\xi_{\text{AVV}}$$

(8) $$\frac{d\xi_{\text{AVV}}}{dt}=\left\{ \begin{array}{c} \left(1-\xi_{\text{AVV}}\right)K_{\text{vo},\text{AVV}}\left(P_{A}-P_{V}\right),P_{A}\geq P_{V}\\ \xi_{\text{AVV}}K_{\text{vc},\text{AVV}}\left(P_{A}-P_{V}\right),P_{A}<P_{V} \end{array}\right.$$

where *B*_AVV_ and *L*_AVV_ are the Bernoulli resistance and the blood inertance, respectively, which depend on the effective length, *l*_eff,AVV_, and effective area, *A*_eff,AVV_. The effective area depends on the annulus area *A*_ann,AVV_ and the state of the valve *ξ*_AVV_, which is 0 when completely closed and 1 when completely open. The valve opens with a rate coefficient of *K*_vo,AVV_ when the atrial pressure is greater than the ventricular pressure and closes with a rate coefficient of *K*_vc,AVV_ when the ventricular pressure is greater than the atrial pressure.

Blood flow in the ventricle is governed by Eqs. (9), (10), (11), (12), (13) and blood flow across the AoV is governed by Eqs. (14), (15), (16), which are equivalent to the equations described above for the atrium and AVV, respectively,

(9) $$\frac{dV_{V}}{dt}=Q_{\text{AVV}}-Q_{\text{AAo}}$$

(10) $$P_{V}=E_{V}\left(V_{V}-V_{0,V}\right)\left(1-K_{s,V}Q_{\text{Ao}}\right)$$

(11) $$E_{V}=k_{V}\frac{g_{1,V}}{1+g_{1,V}}\frac{1}{1+g_{2,V}}+E_{\min,V}$$

(12) $$g_{1,V}={\left(\frac{t-t_{\text{onset},V}}{\tau_{1,V}}\right)}^{m_{1,V}},g_{2,V}={\left(\frac{t-t_{\text{onset},V}}{\tau_{2,V}}\right)}^{m_{2,V}}$$

(13) $$k_{V}=\frac{E_{\max,V}-E_{\min,V}}{\max\left(\frac{g_{1,V}}{1+g_{1,V}}\frac{1}{1+g_{2,V}}\right)}$$

(14) $$\frac{dQ_{\text{AAo}}}{dt}=\frac{1}{L_{\text{AoV}}}\left(P_{V}-P_{\text{AAo}}-B_{\text{AoV}}\left|Q_{\text{AAo}}\right|Q_{\text{AAo}}\right)$$

(15) $$L_{\text{AoV}}=\frac{\rho l_{\text{eff},\text{AoV}}}{A_{\text{eff},\text{AoV}}},\;B_{\text{AoV}}=\frac{\rho }{2A_{\text{eff},\text{AoV}}^{2}},\;A_{\text{eff},\text{AoV}}=A_{\text{ann},\text{AoV}}\xi_{\text{AoV}}$$

(16) $$\frac{d\xi_{\text{AoV}}}{dt}=\left\{ \begin{array}{c} \left(1-\xi_{\text{AV}}\right)K_{\text{vo},\text{AoV}}\left(P_{V}-P_{\text{AAo}}\right),P_{V}\geq P_{\text{AAo}}\\ \xi_{\text{AV}}K_{\text{vc},\text{AoV}}\left(P_{V}-P_{\text{AAo}}\right),P_{V}<P_{\text{AAo}} \end{array}\right.$$

The AoV opens with a rate coefficient of *K*_vo,AoV_ when the ventricular pressure is greater than the aortic pressure and closes with a rate coefficient of *K*_vc,AoV_ when the aortic pressure is greater than the ventricular pressure.

#### 2.2.2 One-dimensional model vessels: aorta and TCPC

Pulsatile blood flow in the aorta and TCPC were modelled using the 1-D equations of conservation of mass (Eq. (17)) and linear momentum (Eq. (18)) applied to an isothermal, incompressible Newtonian fluid flowing in laminar regime inside an impermeable, deformable, tubular 1-D model vessel [25,28],

(17) $$\frac{\partial A}{\partial t}+\frac{\partial \left(UA\right)}{\partial x}=0$$

(18) $$\frac{\partial U}{\partial t}+U\frac{\partial U}{\partial x}=-\frac{1}{\rho }\frac{\partial P}{\partial x}-\frac{22\pi \mu U}{\rho A}$$

where *A*(*x*,*t*) is the luminal area, *U*(*x*,*t*) is the blood flow velocity, *P*(*x*,*t*) is the blood pressure, and *x* is the axial coordinate. The density and viscosity of blood were assumed to be *ρ* = 1060 kg/m^3^ and *μ* = 0.0035 Pa s, respectively.

The set of equations was closed by the pressure–area relationship specified in Eq. (19),

(19) $$P=P_{0}+\frac{\beta }{A_{0}}\left(\sqrt{A}-\sqrt{A_{0}}\right),\beta =\frac{4}{3}\sqrt{\pi }Eh$$

where *P*_0_ and *A*_0_ are the diastolic pressure and cross-sectional area, respectively. The parameter *β* is related to the pulse wave velocity (PWV), *c*, as per Eq. (20),

(20) $$c^{2}=\frac{\beta }{2\rho A_{0}}\sqrt{A}$$

The PWV was assumed to be constant for all the 1-D model vessels of the upper aorta (*c*_Ao_) and TCPC (*c*_TCPC_).

#### 2.2.3 Zero-dimensional models of the upper and lower body

Blood flow in the upper and lower body was modelled based on a model previously used to simulate peripheral vascular beds [29]. This model consists of the resistances to blood flow from arteries (*R*_a_), capillaries (*R*_c_), and veins (*R*_v_), and the compliances of arteries (*C*_a_) and veins (*C*_v_). Using the fluid–electricity analogy, time-varying arterial and venous pressures (*P*_a_(*t*) and *P*_v_(*t*)) and arterial and venous flows (*Q*_a_(*t*) and *Q*_v_(*t*)) in the upper and lower bodies are described through Eqs. (21), (22), (23), (24), (25) and (25), (26), (27), (28), respectively,

(21) $$\frac{dP_{a1}}{dt}=\frac{1}{C_{a1}}\left(Q_{a1}+Q_{a2}+Q_{a3}-Q_{c1}\right)$$

(22) $$Q_{a1}=\frac{P_{\text{BCA}}-P_{a1}}{R_{a1}},\;Q_{a2}=\frac{P_{\text{LCCA}}-P_{a1}}{R_{a2}},\;Q_{a3}=\frac{P_{\text{LSA}}-P_{a1}}{R_{a3}},\;Q_{c1}=\frac{P_{a1}-P_{v1}}{R_{c1}}$$

(23) $$\frac{dP_{v1}}{dt}=\frac{1}{C_{v1}}\left(Q_{c1}-Q_{v1}\right)$$

(24) $$Q_{v1}=\frac{P_{v1}-P_{\text{SVC}}}{R_{v1}}$$

(25) $$\frac{dP_{a2}}{dt}=\frac{1}{C_{a2}}\left(Q_{a4}-Q_{c2}\right)$$

(26) $$Q_{a4}=\frac{P_{\text{DAo}}-P_{a2}}{R_{a4}},Q_{c2}=\frac{P_{a2}-P_{v2}}{R_{c2}}$$

(27) $$\frac{dP_{v2}}{dt}=\frac{1}{C_{v2}}\left(Q_{c2}-Q_{v2}\right)$$

(28) $$Q_{v2}=\frac{P_{v2}-P_{\text{IVC}}}{R_{v2}}$$

where subscripts *R*_a1_, *R*_a2_, *R*_a3_ and *R*_a4_ indicate the arterial resistance to blood flow connected to the BCA, LCCA, LSA, and DAo, respectively; *R*_c1_ and *R*_c2_ indicate the capillary resistance to blood flow in the upper and lower bodies, respectively; *R*_v1_ and *R*_v2_ indicate the venous resistance to blood flow connected to SVC and IVC, respectively. The parameters *C*_a1_ and *C*_a2_ indicate the compliance of arteries in the upper and lower bodies, respectively; and *C*_v1_ and *C*_v2_ indicate the compliance of veins in the upper and lower bodies. The variables *P*_a1_(*t*) and P_a2_(*t*) stand for the arterial pressures in the upper and lower bodies, respectively, and *P*_v1_(*t*) and *P*_v2_(*t*) are the venous pressures in the upper and lower bodies, respectively. The variables *Q*_a1_(*t*), *Q*_a2_(*t*), *Q*_a3_(*t*), *Q*_a4_(*t*), *Q*_c1_(*t*), *Q*_c2_(*t*), *Q*_v1_(*t*) and *Q*_v2_(*t*) are the flows through the resistances with the same subscripts. *P*_BCA_(*t*), *P*_LCCA_(*t*), *P*_LSA_(*t*), *P*_SVC_(*t*), and *P*_IVC_(*t*) are the pressures at BCA, LCCA, LSA, SVC, and IVC, respectively.

#### 2.2.4 Zero-dimensional lung model

Blood flow in the lungs was modelled using Eqs. (29), (30),

(29) $$P_{\text{RPA}}-P_{A}=Q_{\text{RPA}}R_{\text{RPA}}$$

(30) $$P_{\text{LPA}}-P_{A}=Q_{\text{LPA}}R_{\text{LPA}}$$

with *R*_RPA_ and *R*_LPA_ the resistances to blood flow in the RPA and LPA, respectively, *Q*_RPA_(*t*) and *Q*_LPA_(*t*) the blood flow through the RPA and LPA, respectively, and *P*_RPA_(*t*), *P*_LPA_(*t*), and *P*_A_(*t*) the pressures in the RPA, LPA, and atrium, respectively.

### 2.3 Parameter estimation

**Fig. 3:**
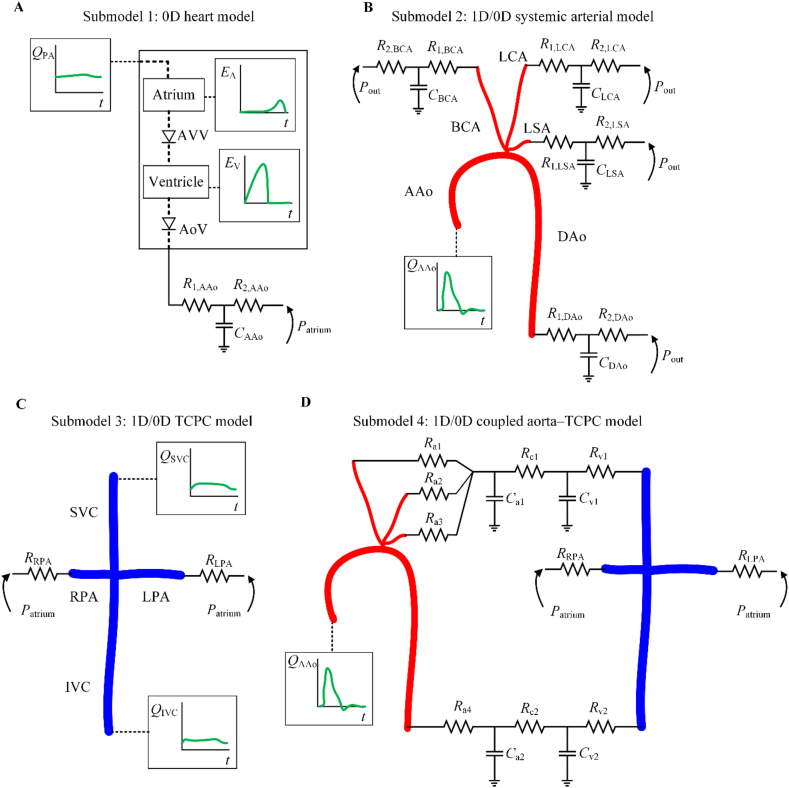
Submodels used in the stepwise parameter estimation methodology. ***A***: Heart model. ***B***: Systemic arterial model. ***C***: Total cavopulmonary connection (TCPC) model. ***D***: Coupled aorta–TCPC model. AAo: ascending aorta; AoV: aortic valve; AVV: atrioventricular valve; BCA: brachiocephalic artery; DAo: descending aorta; IVC: inferior vena cava; LCCA: left common carotid artery; LPA: left pulmonary artery; PA: pulmonary arteries; RPA: right pulmonary artery; SVC: superior vena cava. The remaining model parameters are described in Tables S1–S4 in online-only Data Supplement.

A novel physics-based stepwise methodology involving several simpler models (referred to as ‘submodels’) than the final model shown in Fig. 2 was followed to estimate model parameters. This is detailed in Supplementary Material Section 1**.** The MATLAB code used to implement this methodology is available at https://zenodo.org/records/11076659. The complete, closed-loop model (Fig. 2) was decomposed into four open-loop submodels (Fig. 3). Tables S1–S4 in Supplementary Material show the input data required to estimate each parameter of every submodel.

In Submodel 1, the 0-D univentricular heart is coupled to the pulmonary arterial flow and a three-element Windkessel (3WK) model describing the entire systemic circulation (Fig. 3A). In Submodel 2, the aortic arch and supra-aortic arteries are simulated using the 1-D formulation, downstream vasculatures are described using 3WK models, and ventricular flow into the aorta is prescribed (Fig. 3B). In Submodel 3, a 1-D model of the TCPC is created by prescribing the IVC and SVC flows and modelling the downstream vasculature using pulmonary resistances and a constant atrial pressure (Fig. 3C). In Submodel 4, the systemic arterial (Submodel 2) and TCPC (Submodel 3) models are coupled to form an open loop, where aortic flow is prescribed and the pulmonary arteries are connected to the pulmonary resistances and atrial pressure (Fig. 3D).

Figure S4 in Supplementary Material shows a schematic representation of the closed-loop model for the specific patient analysed in this study, which differs from the generic model shown in Fig. 2. First, there are only two supra-aortic vessels (LSA is missing). Second, the 1-D model LPA segment was divided into two segments to describe a reduction in the LPA calibre due to the compression force exerted by the aorta.

### 2.4 Numerical solution

The set of equations described above was solved numerically, for the complete model and four submodels. The 1-D model equations were solved using a finite element scheme with an explicit second-order Adams-Bashforth scheme to advance in time [28]. The initial conditions for the simulations consisted of zero velocity and uniform pressure equal to the diastolic pressure obtained from the measured LPCW pressure (in the case of Submodel 2 the diastolic pressure at the DAo was used). Nonlinear terms were iteratively solved at each time step using the Newton-Raphson method. The 0-D model equations were solved using the fourth order Runge-Kutta method. All simulations were run on a 4-core Intel® Core™ i5-5200U central processing unit with a clock-speed of 3.2 GHz. Each simulation took around 15 cardiac cycles to reach a periodic solution in about 30 min.

### 2.5 Error calculations

The relative point-to-point error (RPPE, $\varepsilon_{P}$ and $\varepsilon_{Q}$) and the relative error in the mean value ($\varepsilon_{\bar{P}}$ and $\varepsilon_{\bar{Q}}$, Eq. (31)) were used to quantify the accuracy of the pressure (*P*) and flow (*Q*) waves simulated by the models,

(31) $$\varepsilon_{P}=\frac{1}{N}\sum_{i=1}^{N}\left|\frac{P_{i}^{\text{sim}}-P_{i}^{\text{meas}}}{P_{i}^{\text{meas}}}\right|,\;\varepsilon_{\bar{P}}=\frac{{\bar{P}}^{\text{sim}}-{\bar{P}}^{\text{meas}}}{{\bar{P}}^{\text{meas}}},\;\varepsilon_{Q}=\frac{1}{N}\sum_{i=1}^{N}\left|\frac{Q_{i}^{\text{sim}}-Q_{i}^{\text{meas}}}{\max\left(Q^{\text{meas}}\right)}\right|,\;\varepsilon_{\bar{Q}}=\frac{{\bar{Q}}^{\text{sim}}-{\bar{Q}}^{\text{meas}}}{\max\left(Q^{\text{meas}}\right)}$$

where *N* is the total number of samples in a cardiac cycle, the subscript *i* indicates the sample point (sampling rate was 1 kHz), superscripts ‘sim’ and ‘meas’ refer to the simulated and measured, respectively, pressures or flows, the operator max(·) calculates the maximum value of the input variable in a cardiac cycle, and bars indicate mean value of the input variable in a cardiac cycle [30]. Equation (32) was used to compute the relative errors in ventricular *P* and volume (*V*),

(32) $$\varepsilon_{P}=\frac{1}{N}\sum_{i=1}^{N}\left|\frac{P_{i}^{\text{sim}}-P_{i}^{\text{meas}}}{\max\left(P^{\text{meas}}\right)}\right|,\;\varepsilon_{\bar{P}}=\frac{{\bar{P}}^{\text{sim}}-{\bar{P}}^{\text{meas}}}{\max\left(P^{\text{meas}}\right)},\;\varepsilon_{V}=\frac{1}{N}\sum_{i=1}^{N}\;\left|\frac{V_{i}^{\text{sim}}-V_{i}^{\text{meas}}}{V_{i}^{\text{meas}}}\right|,\;\varepsilon_{\bar{V}}=\frac{{\bar{V}}^{\text{sim}}-{\bar{V}}^{\text{meas}}}{{\bar{V}}^{\text{meas}}}$$

### 2.6 Cardiovascular index calculations

The stroke volume (SV, ml/cycle) was calculated as the integral of the aortic flow over time for one cardiac cycle. The heart rate (HR, cycles/min) was calculated as HR = 60/*T*, where *T* is the cardiac cycle period in seconds. From these, the cardiac output (CO, l/min) can be found as CO=SV HR/1000. Moreover, the systemic vascular resistance (SVR, mmHg·min/l) and the pulmonary vascular resistance (PVR, mmHg·min/l) were calculated using Eqs. (33), (34):

(33) SVR=(meanarterialpressure−meanatrialpressure)/CO

(34) PVR=(meanpulmonaryarterialpressure−meanatrialpressure)/CO.

### 2.7 Clinically relevant interventions

Pulmonary vasodilation is a potential pharmacological treatment of Fontan failure, offering clear benefits in patients with markedly high PVR [31]. Even in patients with no or minor increases in PVR, beneficial effects of pulmonary vasodilation have been reported [32]. The degree of patient-specific reduction in pulmonary resistances depends on vasodilator dosage and patient response. This intervention was simulated by reducing the resistances $R_{\text{RPA}}$ and $R_{\text{LPA}}$ downstream the RPA and LPA, respectively, by 25 %.

The potential benefits of a fenestration in the TCPC were investigated. A fenestration allows a portion of blood flow in the IVC to bypass the pulmonary circulation and directly enter the heart through the atrium. The fenestration was modelled as a 3-cm-long, 4-mm-diameter tube with a resistance of 0.5 mmHg s/ml connecting the middle point of the IVC to the atrium. These dimensions were based on the study by Albal et al. [33].

## 3 Results

### 3.1 Baseline fontan circulation

**Fig. 4:**
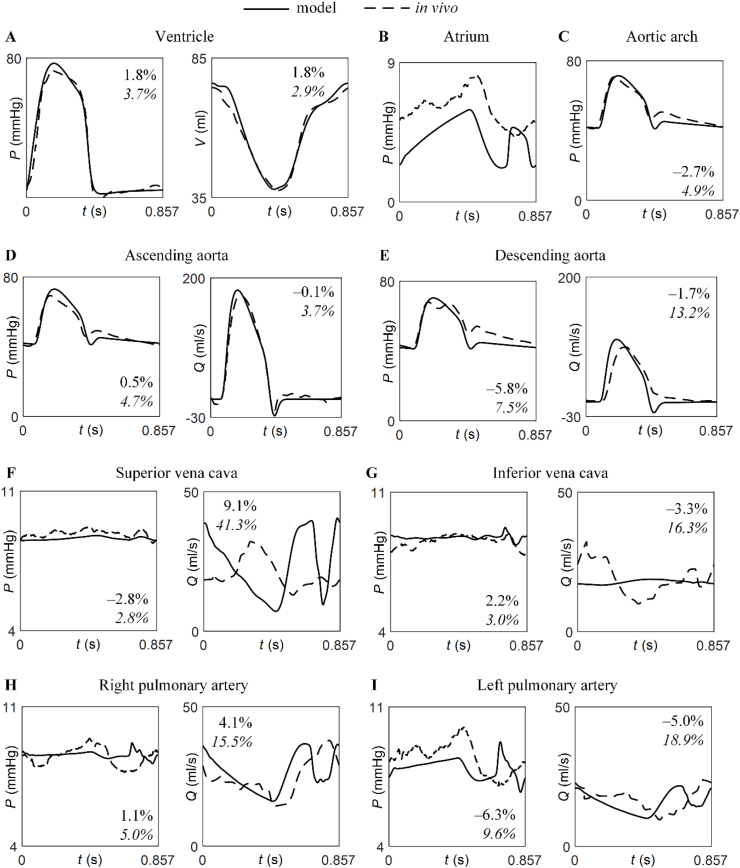
Simulated vs. *in vivo* haemodynamic quantities at baseline. Time-varying pressure (*P*) and volume (*V*) in the ventricle (***A***), *P* in the atrium (***B***) and aortic arch (***C***), and *P* and blood flow (*Q*) waveforms at ascending aorta (***D***), descending aorta (***E***), SVC (***F***), IVC (***G***), RPA (***H***), and LPA (***I***), simulated by the Fontan model (solid lines) and measured *in vivo* (dashed lines). Each panel shows the error in the mean value (top, in regular) and the RPPE (bottom, in italics). No error was computed for the atrium because the *in vivo* measurement corresponds to the LPCW pressure.

The model was able to simulate pulsatile blood pressure in the ventricle (Fig. 4A), atrium (Fig. 4B), aortic arch (Fig. 4C), ascending aorta (Fig. 4D), right pulmonary artery (Fig. 4H), and venae cavae (Fig. 4F and G), with RPPEs smaller than 5 %, except for the descending aorta (7.5 %) (Fig. 4E) and left pulmonary artery (9.6 %) (Fig. 4I). RPPEs in blood flow were considerably larger (<19 %) at the same locations in the descending aorta (Fig. 4E), pulmonary arteries (Fig. 4H and I), and inferior vena cava (Fig. 4G), and notably larger in the superior vena cava (41 %) (Fig. 4F). In the ascending aorta, however, a smaller RPPE in blood flow than in pressure was obtained (3.7 % vs. 4.7 %) (Fig. 4D). The RPPE in ventricular volume was also slightly smaller compared to the RPPE in ventricular pressure (2.9 % vs. 3.7 %) (Fig. 4A). Relative errors in mean blood pressure and flow were considerably smaller than the corresponding RPPEs: average relative errors in mean pressure and flow were 2.9 % and 3.6 %, respectively, whereas the average RPPEs in pressure and flow were 5.2 % and 16.0 %, respectively. Interestingly, these average relative errors were mostly smaller when considering haemodynamic quantities simulated by Submodels 1 to 4 (Table S5, Supplementary Material). In particular, the RPPE for blood flow in the superior vena cava simulated using Submodel 4 was much smaller (18 % vs. 41 %).

Simulated time-varying haemodynamic quantities in the heart chambers and in the arteries and veins of the baseline Fontan model exhibited several characteristic features observed *in vivo*. First, ventricular blood pressure rose rapidly in systole, surpassing aortic pressure during the first half of systole. There was a sharper decrease in ventricular blood pressure in late systole. Ventricular volume decreased during systole and increased during diastole, reaching a minimum at the time of aortic valve closure. In the atrium, blood pressure increased in systole and decreased in diastole, although the model underestimated pressure values by about 2 mmHg and predicted a rise and fall in late diastole that was not observed *in vivo*. Second, along the aorta, systolic ejection produced a sharp rise in pressure followed by a sharp fall. A second, smaller pressure peak was observed at the beginning of diastole, creating an incisura. The ensuing pressure decline was much smoother, although the model underestimated the rate of aortic pressure decay in diastole. Third, the aortic flow wave resembled the aortic pressure waveform but with a region of reversed flow at the time of the incisura and a flatter shape in diastole. Fourth, pressures in the venae cavae remained approximately constant, with similar values in both veins. The model produced similar mean venous flows but failed to capture the *in vivo* wave morphology in both veins. Fifth, in the pulmonary arteries, pressures increased in systole and decreased after aortic valve closure, while the flows decreased in systole and increased after aortic valve closure. However, late-diastolic pressure and flows exhibited peaks and troughs not observed *in vivo*. Similar patterns were observed for the signals simulated by Submodels 1 to 4 (Fig. S5, Supplementary Material), except for pressures and flows held constant in the venae cavae and pulmonary arteries of Submodel 4.

**Fig. 5:**
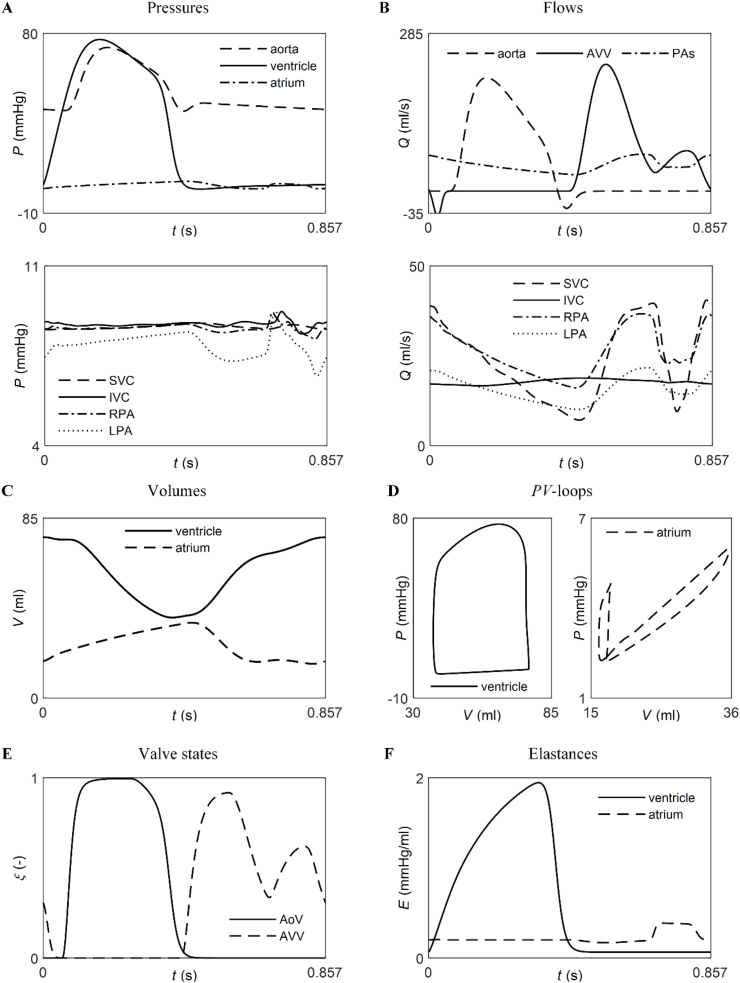
Extended simulated haemodynamic quantities at baseline. Pressures (*P*) and flows (*Q*) at several locations (***A*** and ***B***); ventricular and atrial volumes (*V*) (***C***), *PV*-loops (***D***), and time-varying valve states (*ξ*; fully open when *ξ* = 1 and fully closed when *ξ* = 0) (***E***) and elastance curves (*E*) (***F***). AoV: aortic valve, AVV: atrioventricular valve, IVC: inferior vena cava, LPA: left pulmonary artery, LPCW: left pulmonary capillary wedge, PAs: pulmonary arteries, RPA: right pulmonary artery, SVC: superior vena cava.

Fig. 5 shows direct comparisons of the simulated pressures and flows throughout the Fontan circulation (Fig. 5A and B), ventricular and atrial volumes (Fig. 5C), ventricular and atrial *PV*-loops (Fig. 5D), states of the atrioventricular and aortic valves (Fig. 5E), and ventricular and atrial elastance curves (Fig. 5F). In early systole, ventricular pressure exceeds that in the aorta, causing the aortic valve to gradually open, increasing aortic blood flow, and reducing ventricular volume. By mid-systole, aortic pressure surpasses ventricular pressure, resulting in a gradual closure of the aortic valve, decreased aortic flow, and further reduction in ventricular volume. During early diastole, the atrioventricular valve—which remained closed during systole allowing atrial volume to increase—opens as atrial pressure rises above ventricular pressure. As a result, blood flows through the valve, increasing ventricular volume and decreasing atrial volume. The flow through the atrioventricular valve exhibits two characteristic peaks yielding an atrial *PV*-loop with two loops. This is the result of the partial closure of the atrioventricular valve when flow decreases at the end of passive filling and subsequently wider opening with atrial contraction triggered by a surge in atrial elastance. The atrioventricular valve closes at the end of diastole, allowing the atrium to start filling again. Flow through the aortic and atrioventricular valves have similar shapes to their corresponding valve states. Pressures in the TCPC vessels are approximately constant throughout the cardiac cycle (see scale from 4 to 11 mmHg), while flows decrease in systole and increase in diastole (except for the inferior vena cava), following the pattern of atrioventricular valve flow. There is preferential flow towards the RPA due to the smaller size of the LPA.

The parameter values for the baseline model are tabulated in Table 2, Table 3. In total, the patient-specific model comprises 70 parameters; 45 (64.3 %) were directly estimated from the patient's data, while the remaining 25 (35.7 %) were manually adjusted. These manually adjusted parameters include those describing the elastance functions of the atrium and ventricle, as well as the opening and closing of the atrioventricular and aortic valves. Parameter values for all submodels are provided in Tables S1–S4 (Supplementary Material).

### 3.2 Clinically relevant interventions

**Fig. 6:**
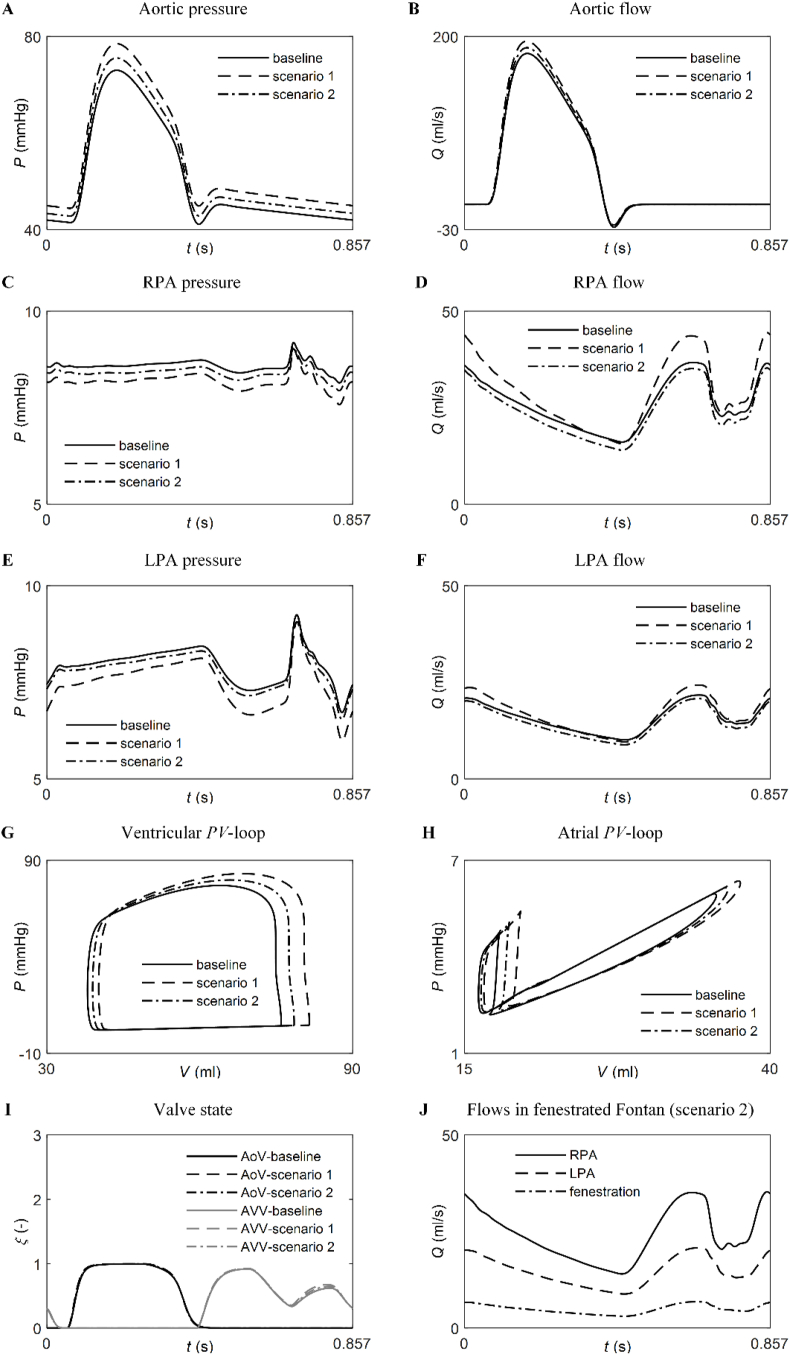
Simulated time-varying haemodynamic quantities at baseline, with pulmonary vasodilator (Scenario 1), and fenestration opening (Scenario 2): Aortic pressure (***A***) and flow (***B***), RPA pressure (***C***) and flow (***D***), LPA pressure (***E***) and flow (***F***), ventricular and atrial *PV*-loops (***G*** and ***H***, respectively), valve states (***I***), and flows in fenestrated Fontan circulations (Scenario 2 only) (***J***). AoV: aortic valve, AVV: atrioventricular valve, LPA: left pulmonary artery, RPA: right pulmonary artery.

Fig. 6 compares simulated haemodynamic quantities at baseline with hypothetical clinical scenarios: pulmonary vasodilation (Scenario 1) and atrial fenestration opening in the TCPC (Scenario 2). Table 4 presents several cardiovascular indices to quantitatively compare these circulations. The simulated scenarios allow for exploring the likely changes in patient-specific haemodynamics resulting from the interventions. In Scenario 1, a 25 % reduction in pulmonary vascular resistance led to an increase in mean pulmonary blood flow in the RPA (+12.0 %, Fig. 6D) and LPA (+6.8 %, Fig. 6F). Consequently, the preload of the single-ventricle, as measured by ventricular end-diastolic pressure, increased (+8.4 %, Fig. 6G), resulting in a larger SV (+10 %) and increased CO (+10 %) (Fig. 6B). This simulation suggests that pulmonary vasodilation could be a potentially beneficial therapy for the patient studied.

**Table 4:** Cardiovascular characteristics of the baseline, Scenario 1, and Scenario 2 circulations. In Scenarios 1 and 2, the relative percentage increase/decrease with respect to baseline is included. SV: stroke volume, CO: cardiac output, SVR: systemic vascular resistance, PVR: pulmonary vascular resistance, RPA: right pulmonary artery, LPA: left pulmonary artery.

	Baseline	Scenario 1	Scenario 2
SV (ml/cycle)	35.72	39.31 (+10 %)	37.39 (+4.7 %)
CO (l/min)	2.50	2.75 (+10 %)	2.62 (+4.8 %)
Systolic arterial pressure (mmHg)	72.99	78.50 (+7.5 %)	75.54 (+3.5 %)
Mean arterial pressure (mmHg)	50.66	54.51 (+7.6 %)	52.43 (+3.5 %)
Mean pulmonary arterial pressure (mmHg)	8.25	7.82 (−5.2 %)	8.10 (−1.8 %)
Mean atrial pressure (mmHg)	4.11	4.42 (+7.5 %)	4.25 (+3.4 %)
Ventricular end-diastolic pressure (mmHg)	4.25	4.61 (+8.4 %)	4.42 (+3.5 %)
Ventricular peak pressure (mmHg)	72.74	83.10 (+14.2 %)	79.80 (+9.7 %)
Mean TCPC pressure (mmHg)	8.58	8.06 (−6.1 %)	8.29 (−3.4 %)
SVR (mmHg·min/l)	18.62	18.20 (−2.2 %)	18.41 (−1.1 %)
PVR (mmHg·min/l)	1.66	1.23 (−26.0 %)	1.47 (−11.4 %)
Mean RPA flow (ml/s)	25.98	29.11 (+12.0 %)	24.14 (−7.1 %)
Mean LPA flow (ml/s)	15.68	16.75 (+6.8 %)	14.59 (−6.9 %)

The opening of a fenestration (Scenario 2) also increased the preload to the ventricle (Fig. 6G), but to a lesser extent compared to Scenario 1 (+3.5 % vs. 8.4 %), resulting in a smaller increase in SV and CO (+4.8 % vs. 10 %) (Fig. 6B). However, pulmonary blood flow decreased (by −7.1 % in the RPA and −6.9 % in the LPA) (Fig. 6D and F), as the fenestration flow exceeded the net increase in CO (0.3 l/min vs. 0.12 l/min, respectively). Opening the fenestration has another drawback: it reduces the oxygen saturation of systemic arterial blood (beyond the scope of the present study).

With both interventions, the model predicted changes in the values of blood pressures (Fig. 6A–C and E), blood flows (Fig. 6B–D and F), and *PV*-loops (Fig. 6G and H) throughout the Fontan circulation, but not in the morphologies of these haemodynamic quantities compared to their corresponding baseline values. Aortic and atrioventricular valve states remained unchanged (Fig. 6I). Lastly, the morphology of the fenestration flow wave decreased in systole, increased in diastole, and featured a trough in late diastole, following a similar pattern to the morphology of simulated pulmonary flows (Fig. 6J).

## 4 Discussion

We have demonstrated the feasibility of creating an accurate, subject-specific, 1-D/0-D model of pulsatile blood flow in the Fontan circulation using clinical data from combined cardiac catheterisation and cardiac magnetic resonance exams (i.e., an iCMR exam). Previous models have shown promise in contributing to the optimal management of other congenital heart diseases [34,35]. Our model could enhance the state-of-the-art assessment of Fontan haemodynamics provided by iCMR exams by (i) augmenting the data with flow and pressure signals at locations where no *in vivo* signals are available, and (ii) providing a mechanistic understanding of how different haemodynamic measurements relate to each other, both at the time of the exam and in virtual clinical scenarios. For example, the model could be used to virtually study a patient's response to clinical interventions, such as pulmonary vasodilator administration or atrial fenestration opening.

Compared to other studies using reduced-order modelling [18], our 1-D/0-D model of the Fontan circulation has fewer parameters and can be calibrated on a patient-specific basis. About two-thirds of the model parameters can be directly calculated from *in vivo* iCMR data. This is achieved by using (i) the 1-D formulation for the upper aorta, supra-aortic vessels, and TCPC, where geometric and mechanical properties can be obtained from the patient's anatomical and haemodynamic measurements, and (ii) 0-D modelling for the contraction and filling of the univentricular heart and other blood vessels where imaging and haemodynamic data are not available. The remaining one-third of the model parameters describe the elastance functions of the atrium and ventricle, and the opening and closing of the atrioventricular and aortic valves. These parameters cannot be directly measured *in vivo* due to their mathematical nature (e.g. time and rate constants describing the elastance curves in Eqs. (4) and (12) or the lack of detailed haemodynamic measurements that are currently not obtained during iCMR exams (e.g. blood pressure and flow around the aortic and atrioventricular valves). Instead, they require manual adjustment to match measured aortic flow wave and ventricular pressure and volume.

The relative errors in the simulated haemodynamic signals simulated by our model are comparable to those observed in 1-D/0-D models of the normal adult circulation (see Table 4 in Ref. [25]), with overall smaller relative errors for pressures compared to flows. This likely results from all parameter estimation methods used to calibrate the model emphasising the minimisation of errors in pressure signals. Furthermore, relative errors in mean values are smaller than point-to-point errors, as quantified by the mean square metric, indicating the model's superior ability to estimate mean haemodynamic quantities described by simple algebraic equations (e.g. Eq. (22)) compared to pulsatile-induced variations in haemodynamic signals described by differential equations (e.g. Eqs. (17), (18)). Additionally, ensuring proper alignment of flow and pressure signals in time during model calibration is crucial due to the asynchronous nature of iCMR data acquisition [36]. This limitation of the iCMR procedure can introduce inconsistencies in the data (e.g. in heart rate and mean volume flow rates), which may partly explain errors in simulated haemodynamic quantities. Therefore, it is essential to analyse uncertainties affecting the measured data before translating the model to clinical practice.

Our model can augment data from iCMR exams by simulating clinically relevant interventions that change haemodynamic properties of the Fontan circulation, such as geometrical and material properties. This is possible because our model's design assigns clear physical meaning to all parameters. Consequently, we investigated two virtual interventions —pulmonary vasodilation and atrial fenestration opening— for which distinguishing responders from non-responders *in vivo* is challenging. For the patient in this study, both interventions increased ventricle preload and, hence, cardiac output via the Frank-Starling mechanism, with greater effects observed with pulmonary vasodilation. These exemplar virtual interventions illustrate the model's ability to compare the predicted haemodynamic efficacy of different treatments. For instance, increased cardiac output results in increased blood flow towards the TCPC, potentially improving overall haemodynamic equilibrium. However, whether maintaining, closing, or reopening a fenestration can achieve better haemodynamic equilibrium in Fontan failure patients likely depends on an individual's haemodynamics and remains to be elucidated [37]. The decrease in pulmonary blood flow predicted by our model in the studied patient with a simulated fenestration will likely result in impaired oxygen delivery to the body. Therefore, our model suggests that opening a fenestration might not improve the patient's haemodynamics, making it a less favourable therapeutic option.

These findings are consistent with those of Puelz et al. [18] which also demonstrated an increase in cardiac output and a decrease in pulmonary artery pressure and flow compared to baseline Fontan models calibrated with literature data. The magnitude of these changes was similar between the studies: our model showed a 5 % increase in cardiac output versus approximately 10 % in Puelz et al.’s 1-D/0-D model, a 2 % decrease in pulmonary artery pressure versus approximately 4 %, and a 14 % decrease in pulmonary artery flow versus approximately 7 %. While their model allowed for pressure and flow in more locations (including 150 1-D model vessels and several 0-D model organ beds), calibrating its parameters on a patient-specific basis was challenging [18]. In contrast, our simpler model enables calibration using patient-specific data.

In addition to the two virtual clinical scenarios studied here, our model could also aid in identifying potential mechanisms of Fontan failure for a specific patient, including myocardial dysfunction, altered resistance/compliance in the pulmonary or systemic vascular beds, and reduced systemic/pulmonary venous return. Furthermore, while this study assessed a patient with hypoplastic left heart syndrome, other conditions related to the Fontan circulation (e.g. tricuspid atresia) could also be analysed using our physics-based model.

This study has several limitations. First, clinical interventions should be performed on both the model and the patient to test the model's predictive ability. This would involve taking haemodynamic measurements at baseline and with each intervention. Second, the model does not incorporate baroreceptor feedback present *in vivo*, which typically regulates properties such as resistances affecting cardiac output and blood pressure. Third, the model and calibration method have only been verified in one subject. Therefore, further verification in a clinical cohort—with different configurations of the supra-aortic vessels and TCPC, and considering collateral flows present in some patients—is essential before the computational approach can become a clinical tool for assessing the Fontan circulation.

## 5 Conclusion

We have demonstrated the feasibility and potential clinical utility of subject-specific, closed-loop, 1-D/0-D modelling of pulsatile haemodynamics in the Fontan circulation, calibrated using haemodynamic data acquired through combined cardiac catheterisation and magnetic resonance exams. The model offers a mechanistic understanding of the physical relationships between different measurements and allows for virtual testing of the Fontan circulation's response to clinical interventions, potentially optimising clinical management and guiding further research in the Fontan population. Future efforts will concentrate on broadening the model's applicability to a wider range of patient populations and clinical scenarios.

## Ethical approval

This study complied with the 1964 Declaration of Helsinki and was approved by the London Westminster Research Ethics Committee, London UK. Ethics Number: 09H0804062.

## Data availability statement

Data included in article/supplementary material/referenced in article.

## CRediT authorship contribution statement

**Jorge Aramburu:** Writing – original draft, Visualization, Validation, Software, Methodology, Investigation, Funding acquisition, Formal analysis, Data curation, Conceptualization. **Bram Ruijsink:** Writing – review & editing, Resources, Data curation. **Radomir Chabiniok:** Writing – review & editing, Supervision, Methodology, Conceptualization. **Kuberan Pushparajah:** Writing – review & editing, Supervision, Resources, Project administration, Funding acquisition, Data curation, Conceptualization. **Jordi Alastruey:** Writing – review & editing, Writing – original draft, Supervision, Project administration, Methodology, Funding acquisition, Formal analysis, Conceptualization.

## Declaration of competing interest

The authors declare the following financial interests/personal relationships which may be considered as potential competing interests: Jorge Aramburu reports financial support was provided by Ministerio de Educación, Cultura y Deporte (Spanish Government). Jordi Alastruey reports financial support was provided by 10.13039/501100000266Engineering and Physical Sciences Research Council. If there are other authors, they declare that they have no known competing financial interests or personal relationships that could have appeared to influence the work reported in this paper.
