# Supplementary material for: Patient-specific closed-loop model of the fontan circulation: Calibration and validation
Source: Heliyon. 2024 Apr 26;10(9):e30404. doi: 10.1016/j.heliyon.2024.e30404 (PMC11089314; doi:10.1016/j.heliyon.2024.e30404)
Supplement: Multimedia component 1 [file mmc1.pdf]

Online Supplement

Patient-specific closed-loop model of the Fontan circulation: calibration and validation

Jorge Aramburu,[1] Bram Ruijsink,[2] Radomir Chabiniok,[2, 3] Kuberan Pushparajah,[2, 4] Jordi Alastruey[2, *]

[1] Universidad de Navarra, TECNUN Escuela de Ingeniería, Pº Manuel Lardizabal 13, 20018 Donostia/San Sebastián, Spain.

[2] School of Biomedical Engineering and Imaging Sciences, King's College London, St Thomas' Hospital, SE1 7EH, London, UK.

[3] Division of Pediatric Cardiology, Department of Pediatrics, UT Southwestern Medical Center, Dallas, TX.

[4] Department of Congenital Heart Disease, Evelina Children's Hospital, SE1 7EH, London, UK.

*Corresponding author:

Address: Biomedical Engineering, 3rd Floor, Lambeth Wing, St Thomas' Hospital, 249 Westminster Bridge Rd, Lambeth, London, SE1 7EH, UK

Telephone number: +44 (0)20 7848 9556

Email: jordi.alastruey-arimon@kcl.ac.uk

1 Physics-based parameter estimation method

This appendix describes in detail the physics-based parameter estimation method. The general stepwise methodology for estimating the parameters of the closed-loop model in **Fig. 2** is explained in Section 1.1. It involves the four submodels illustrated in **Fig. 3**. The parameter estimation method is based on the minimization of a cost function using the Newton–Raphson method, which is described in Section 1.2. The application of the Newton–Raphson method to five different types of parameter estimation problems—referred to as Parameter Estimation 1 (PE1) to 5 (PE5)—is shown in Sections 1.3–1.6. In all these cases, the solution could be found graphically by plotting the corresponding cost functions, since a maximum of two parameters are estimated for each model. All the parameters of the four submodels are presented in **Table S1** to **Table S4**. Parameters associated with the left subclavian artery (LSA) are not included, since this artery was absent in the patient analysed in this study.

1.1 Parameter estimation methodology

1.1.1 Submodel 1: 0-D heart model

The first step consists of estimating the parameters of Submodel 1 (**Fig. 3A**). To do so, first the parameters of the elastance of the ventricle were defined (Eq. (11) in the main manuscript) from a measured elastance defined using $E(t) = P_V(t)/(V_V(t) - V_{0,V})$, where $P_V(t)$ is the measured ventricular pressure, $V_V(t)$ is the measured ventricular volume, and $V_{0,V}$ is a parameter that was estimated so that the maximum elastance was 2 mmHg/ml. Using the measured elastance, the parameters of the elastance of the ventricle were estimated, by comparing the measured elastance with the simulated elastance: $E_{min,V}$, $E_{max,V}$, $m_{1,V}$, $m_{2,V}$, $\tau_{1,V}$, $\tau_{2,V}$, $t_{onset,V}$. Once the elastance curves were defined, the parameter $K_{S,V}$ was estimated from the measured ascending aortic flow, $Q_{AAo}(t)$,

$$K_{S,V} = \mathrm{PE1}\big(Q_{AAo}(t), V_V(t), E_V(t), V_{0,V}\big), \tag{1}$$

with PE1 the parameter estimation method described in Section 1.3. Then, the parameters of the 3WK model of Submodel 1 ($R_{1,AAo}$, $R_{2,AAo}$, $C_{AAo,}$) were estimated, using

$$\{R_{2,AAo}, C_{AAo}\} = \mathrm{PE3}(Q_{AAo}(t), P_{AAo}(t), P_{atrium}), \tag{2}$$

with PE3 the parameter estimation method described in Section 1.5, $P_{AAo}(t)$ the measured ascending aortic pressure, and P_{atrium} the atrial pressure. For an initial estimate of the parameters

of the 3WK model, P_{atrium} was prescribed as the average measured left pulmonary capillary wedge LPCW pressure. The resistance $R_{1,\mathrm{AAo}}$ was calculated using Eq. (28). The parameter estimation of this 3WK model was repeated, once all the parameters of Submodel 1 were estimated, with P_{atrium} equal to the atrial pressure (instead of the LPCW pressure). Then, the parameters of the aortic valve (AoV) ($\underline{l}_{\mathrm{eff,AoV}}$, $A_{\mathrm{ann,AoV}}$, $K_{\mathrm{vo,AoV}}$, $K_{\mathrm{vc,AoV}}$) were estimated considering a constant inflow of blood to the ventricle (i.e., the average of the measured aortic flow) into the model of the ventricle coupled to the 3WK model. Finally, the parameters of the atrioventricular valve (AVV) ($\underline{l}_{\mathrm{eff,AVV}}$, $A_{\mathrm{ann,AVV}}$, $K_{\mathrm{vo,AVV}}$, $K_{\mathrm{vc,AVV}}$), and atrium ($V_{0,\mathrm{A}}$, $E_{\mathrm{min,A}}$, $E_{\mathrm{max,A}}$, $m_{1,\mathrm{A}}$, $m_{2,\mathrm{A}}$, $\tau_{1,\mathrm{A}}$, $\tau_{2,\mathrm{A}}$, $t_{\mathrm{onset,A}}$) were adjusted by comparing the results of the simulation of Submodel 1 with the *in vivo* measurements.

1.1.2 Submodel 2: 1-D/0-D systemic arterial model

First, the length and luminal diameters of all the 1-D model arterial segments of the systemic circulation (**Fig. 3B**) were measured from medical images. Second, the PWV in each 1-D model segment was estimated by (i) using the *PU*-loop method [1] for the AAo and the DAo segments in which blood pressure and flow velocity data were available, and (ii) taking the average value for all the 1-D model segments in Submodel 2.

Third, the 3WK model parameters in the three distal branches were estimated. First, P_{out}—assumed to be constant in all 3WK models—was estimated by assuming an exponential decay in the aortic pressure during diastole, using the parameter estimation method PE2 described in Section 1.4,

$$\{P_{\mathrm{out}}, \tau\} = \mathrm{PE2}(P_{\mathrm{AAo}}, P_0, t_0). \tag{3}$$

The method PE3 (see Section 1.5) was used to calculate $R_{1,\mathrm{DAo}}$, $R_{2,\mathrm{DAo}}$, and C_{DAo}, from $Q_{\mathrm{DAo}}(t)$, $P_{\mathrm{DAo}}(t)$, and P_{out} through

$$\{R_{2,\mathrm{DAo}}, C_{\mathrm{DAo}}\} = \mathrm{PE3}(Q_{\mathrm{DAo}}(t), P_{\mathrm{DAo}}(t), P_{\mathrm{out}}). \tag{4}$$

Fourth, the remaining parameters ($R_{1,\mathrm{BCA}}$, $R_{2,\mathrm{BCA}}$, C_{BCA}, $R_{1,\mathrm{LCCA}}$, $R_{2,\mathrm{LCCA}}$, and C_{LCCA}) were calculated using the methodology described by Alastruey et al. [2], in which the capacitances and resistances are distributed in the head-and-neck arteries based on distal vessel diameters. If flow and pressure waveforms were known at the BCA and LCCA, the 3WK parameters distal to these arterial segments can also be estimated using PE3.

1.1.3 Submodel 3: 1-D/0-D total cavopulmonary connection (TCPC) model

First, the length and luminal diameters of all the 1-D model arterial segments of the TCPC model (**Fig. 3C**) were measured from medical images. Second, the PWV in each 1-D model segment was calculated using (i) the sum-of-squares method [3] from the pressure and flow measurements at the IVC, SVC, RPA, and LPA, and (ii) taking the average value for all the 1-D model segments in Submodel 3. Third, the RPA and LPA resistances, R_{RPA} and R_{LPA}, were calculated using flow and pressure measurements through Eqs. (29) and (30) in the main manuscript. The average pressure in the atrium, P_{atrium}, has the same value as in Submodel 1.

1.1.4 Submodel 4: 1-D/0-D coupled aorta–TCPC model

In Submodel 4, Submodels 2 and 3 are coupled using the 0-D models of the upper and lower bodies. For the lower body, the arterial resistance and compliance (R_a and C_a) were taken from Submodel 2. Using pressure and flow measurements at the DAo and IVC vessels, the remaining parameters (R_c, R_v, and C_v) were calculated using the parameter estimation methods PE4 (Section 1.6.1) and PE5 (Section 1.6.2).

For the upper body, the simulation results of aortic arch pressure and flow obtained from running Submodel 2 were used to estimate R_a and C_a using PE3 (Section 1.5). This resistance and this compliance were distributed in the head-and-neck arteries using the methodology described in Alastruey et al. [2], where resistances and compliances are distributed according to distal vessel diameters. Then, using pressure and flow measurements at the SVC and the Submodel 2 simulation results at the aortic arch, the remaining parameters (R_c, R_v, and C_v) were calculated using PE4 (Section 1.6.1) and PE5 (Section 1.6.2).

1.1.5 Full closed-loop model

A full closed-loop 1-D/0-D model of the Fontan circulation was obtained by coupling the 0-D heart model (Submodel 1) with the 1-D/0-D coupled aorta–TCPC model (Submodel 4). In this last step, the total blood volume flowing through the system must be defined (V_{blood}). To estimate this parameter, three simulations of the model were run with different values of V_{blood}. The results of these simulations show that both the mean aortic pressure and mean aortic flow linearly depend on V_{blood} (**Fig. S1**), allowing for the calculation of V_{blood} that matches the measured mean aortic flow, and a different V_{blood} that matches the measured mean aortic pressure. The resulting average value from these two values was taken as V_{blood} in the model.

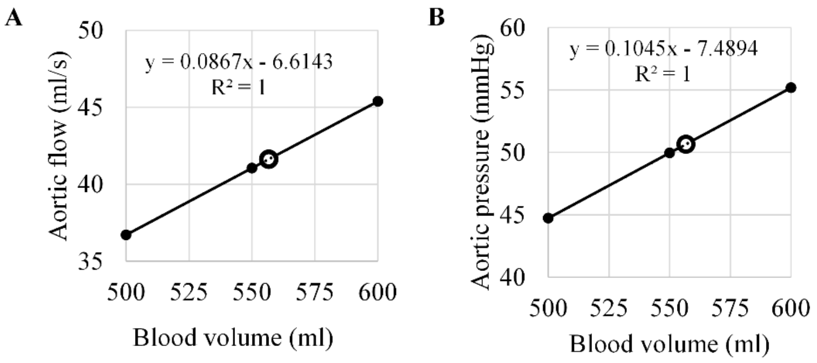

Fig. S1 Dependence of mean aortic flow (***A***) and mean aortic pressure (***B***) with the total blood volume used in the closed-loop model. The results are shown for three different values of blood volume (500, 550, and 600 ml; black circles). From these, a linear relationship was obtained (R^2-value equals 1). White circles indicate the value of blood volume for the measured mean aortic flow and aortic pressure.

1.2 Newton–Raphson method

Let us consider the system given in Eq. (5):

$$y(k) = f(\mathbf{u}(k), \boldsymbol{\theta}) + \varepsilon(k), \tag{5}$$

where $y(k)$ is a measurement at time *k* (i.e., t_k), $\mathbf{u}(k)$ is the known input vector at time *k*, $\boldsymbol{\theta}$ is the vector of dimension *n* of unknown parameters, and $\varepsilon(k)$ is the measurement error at time *k*. To estimate the vector of unknown parameters that best fits the experimental observations, the following model is considered:

$$y(k) = f\left(\mathbf{u}(k), \widehat{\boldsymbol{\theta}}\right) + e(k), \tag{6}$$

where $\widehat{\boldsymbol{\theta}}$ is the vector of dimension *n* of the estimated parameters and $e(k)$ is the residual error at time *k*.

For *m* measurements ($m > n$), Eq. (7) can be built,

$$\mathbf{y} = \mathbf{f}\left(\mathbf{u}, \widehat{\boldsymbol{\theta}}\right) + \mathbf{e},$$
$$\mathbf{y} = \begin{Bmatrix} y(1) \\ \vdots \\ y(m) \end{Bmatrix}, \quad \mathbf{f}\left(\mathbf{u}, \widehat{\boldsymbol{\theta}}\right) = \begin{Bmatrix} f\left(\mathbf{u}(1), \widehat{\boldsymbol{\theta}}\right) \\ \vdots \\ f\left(\mathbf{u}(m), \widehat{\boldsymbol{\theta}}\right) \end{Bmatrix}, \qquad \mathbf{e} = \begin{Bmatrix} e(1) \\ \vdots \\ e(m) \end{Bmatrix} \tag{7}$$

A curve fitting based on the minimization of the squared residual error, consists of minimizing the cost function given by

$$\boldsymbol{\Phi}\left(\widehat{\boldsymbol{\theta}}\right) = \mathbf{e}^{\mathrm{T}}\mathbf{e} = \left[\mathbf{y} - \mathbf{f}\left(\mathbf{u}, \widehat{\boldsymbol{\theta}}\right)\right]^{\mathrm{T}} \left[\mathbf{y} - \mathbf{f}\left(\mathbf{u}, \widehat{\boldsymbol{\theta}}\right)\right] = \sum_{k=1}^{m} \left[y(k) - f\left(\mathbf{u}(k), \widehat{\boldsymbol{\theta}}\right)\right]^2. \tag{8}$$

When minimizing $\boldsymbol{\Phi}\left(\widehat{\boldsymbol{\theta}}\right)$, Eq. (9) must be fulfilled,

$$\frac{\partial \boldsymbol{\Phi}(\widehat{\boldsymbol{\theta}})}{\partial \widehat{\theta}_i} = -2\ \sum_{k=1}^{m} \left[\left[y(k) - f\left(\mathbf{u}(k), \widehat{\boldsymbol{\theta}} \right) \right] \frac{\partial f\left(\mathbf{u}(k), \widehat{\boldsymbol{\theta}} \right)}{\partial \widehat{\theta}_i} \right] = 0, \forall i = 1, 2 \ldots n. \tag{9}$$

The parameters in $\widehat{\boldsymbol{\theta}}$ can be calculated by solving the system of *n* equations and *n* unknowns in Eq. (9).

When the model is not linear, the solution must be calculated iteratively, starting from an initial solution, until a valid solution is obtained. This can be achieved using the Newton–Raphson method. At each iteration of the method, the cost function given by Eq. (8) is approached around the old values of the estimated parameters using a second-order Taylor series approximation, and the new values of the estimated parameters are the values at the minimum of the approached cost function. Mathematically, the second-order Taylor series around the vector of estimated parameters at iteration *i* is given by

$$C_i\left(\widehat{\boldsymbol{\theta}}_i\right) = \boldsymbol{\Phi}\left(\widehat{\boldsymbol{\theta}}_i\right) + \mathbf{q}_i^{\mathrm{T}}\left(\widehat{\boldsymbol{\theta}} - \widehat{\boldsymbol{\theta}}_i\right) + \frac{1}{2}\left(\widehat{\boldsymbol{\theta}} - \widehat{\boldsymbol{\theta}}_i\right)^{\mathrm{T}} \mathbf{H}_i\left(\widehat{\boldsymbol{\theta}} - \widehat{\boldsymbol{\theta}}_i\right), \tag{10}$$

where $\mathbf{q}_i = \partial \boldsymbol{\Phi}\left(\widehat{\boldsymbol{\theta}}\right) / \partial \widehat{\boldsymbol{\theta}}$ is the gradient vector evaluated at $\widehat{\boldsymbol{\theta}}_i$ and $\mathbf{H}_i$ is the Hessian matrix evaluated at $\widehat{\boldsymbol{\theta}}_i$. The element *j,k* of the Hessian matrix is defined by

$$H_{jk}\left(\widehat{\boldsymbol{\theta}}\right) = \frac{\partial^2 \boldsymbol{\Phi}}{\partial \widehat{\theta}_j \partial \widehat{\theta}_k}. \tag{11}$$

If $C_i\left(\widehat{\boldsymbol{\theta}}_i\right)$ is minimized, then the gradient of $C_i\left(\widehat{\boldsymbol{\theta}}_i\right)$ must be the zero vector, i.e.,

$$\frac{\partial C_i}{\partial \widehat{\boldsymbol{\theta}}} = \mathbf{q}_i + \mathbf{H}_i\left(\widehat{\boldsymbol{\theta}} - \widehat{\boldsymbol{\theta}}_i\right) = \mathbf{0}. \tag{12}$$

Equation (12) leads to the following recursive law when the Hessian matrix is a regular matrix,

$$\widehat{\boldsymbol{\theta}}_{i+1} = \widehat{\boldsymbol{\theta}}_i - \mathbf{H}_i^{-1} \mathbf{q}_i. \tag{13}$$

Therefore, in every iteration both the Hessian matrix and the gradient vector must be computed. The convergence of this method is relatively fast. However, the Hessian matrix must be positive-definite, which is not the case if the vector of estimates is far from the minimum.

1.3 Parameter estimation for the ventricle pressure model (PE1)

In addition to the time-varying elastance curve, the ventricle pressure model shown in the main text via Eqs. (9) and (16) in the main manuscript consists of two parameters: $V_{0,\mathrm{V}}$ and $K_{\mathrm{s,V}}$. Assuming that the value of $V_{0,\mathrm{V}}$ is already known, then the parameter to be estimated is $\widehat{\boldsymbol{\theta}} = \widehat{K}_{\mathrm{s,V}}$, and Eq. (6) is given by

$$P_{\mathrm{V}}(t_k) = E_{\mathrm{V}}(t_k)\left[V_{\mathrm{V}}(t_k) - V_{0,\mathrm{V}}\right]\left[1 - \widehat{K}_{\mathrm{s,V}} Q_{\mathrm{AAo}}(t_k)\right] + e(t_k). \tag{14}$$

This is the approached used in our study, with $V_{0,\mathrm{V}}$ calculated using measured ventricular pressure and volume and assuming the maximum elastance, i.e., $\max(P_\mathrm{V}(t)/(V_\mathrm{V}(t) - V_0(t)))$ is 2 mmHg/ml. The first-order and second-order derivatives of the cost function with respect to the estimated parameter are

$$\frac{\partial f}{\partial \hat{K}_\mathrm{s}} = -E_\mathrm{V}(t_k)\left[V_\mathrm{V}(t_k) - V_{0,\mathrm{V}}\right]Q_\mathrm{AAo}(t_k) \tag{15}$$

$$\frac{\partial^2 f}{\partial \hat{K}_\mathrm{s}^2} = 0 \tag{16}$$

This parameter estimation methodology is referred to as PE1; i.e.,

$$\hat{K}_\mathrm{s} = \mathrm{PE1}\left(Q_\mathrm{AAo}(t), V_\mathrm{V}(t), E_\mathrm{V}(t), V_{0,\mathrm{V}}\right). \tag{17}$$

1.4 Parameter estimation for the exponential pressure decay model (PE2)

The exponential pressure decay model consists of three parameters (P_0, P_out, and τ) related through

$$P(t^*) = (P_0 - P_\mathrm{out})e^{-\frac{t^*}{\tau}} + P_\mathrm{out}, \tag{18}$$

where $P_0 = P(t^* = 0)$, $t^* = t - t_0$, with t_0 the time at which the exponential decay starts in the cardiac cycle, and $P_\mathrm{out} = P(t^* \to \infty)$. Assuming that P_0 is known (e.g., from direct observation of a measured aortic pressure waveform), then there are only two parameters to be estimated; i.e., $\hat{\boldsymbol{\theta}} = \{\hat{P}_\mathrm{out}, \hat{\tau}\}$, and Eq. (6) is given by

$$P(t_k^*) = \left(P_0 - \hat{P}_\mathrm{out}\right)e^{-\frac{t_k^*}{\hat{\tau}}} + \hat{P}_\mathrm{out} + e(t_k^*). \tag{19}$$

The first-order and second-order derivatives of the cost function are given by Eqs. (20)–(24),

$$\frac{\partial f}{\partial \hat{P}_\mathrm{out}} = 1 - e^{-\frac{t_k^*}{\hat{\tau}}} \tag{20}$$

$$\frac{\partial f}{\partial \hat{\tau}} = t_k^*\left(P_0 - \hat{P}_\mathrm{out}\right)\hat{\tau}^{-2}e^{-\frac{t_k^*}{\hat{\tau}}} \tag{21}$$

$$\frac{\partial^2 f}{\partial \hat{P}_\mathrm{out}^2} = 0 \tag{22}$$

$$\frac{\partial^2 f}{\partial \hat{\tau}^2} = \frac{t_k^*\left(P_0 - \hat{P}_\mathrm{out}\right)}{\tau^3}\left(\frac{t_k^*}{\hat{\tau}} - 2\right)e^{-\frac{t_k^*}{\hat{\tau}}} \tag{23}$$

$$\frac{\partial^2 f}{\partial \hat{P}_\mathrm{out}\hat{\tau}} = \frac{\partial^2 f}{\partial \hat{\tau}\hat{P}_\mathrm{out}} = -\frac{t_k^*}{\hat{\tau}^2}e^{-\frac{t_k^*}{\hat{\tau}}} \tag{24}$$

This parameter estimation methodology is referred to as PE2; i.e.,

$$\left\{\hat{P}_\mathrm{out}, \hat{\tau}\right\} = \mathrm{PE2}(P(t), P_0, t_0) \tag{25}$$

1.5 Parameter estimation for the three-element Windkessel model (PE3)

3WK models were used in Submodels 1 and 2 (**Fig. S2**). These are defined by the proximal resistance (R_1), the distal resistance (R_2), the capacitance (C), and the distal pressure (P_{out}). The relation between the flow and the pressure (Q_{in} and P_{in}, respectively) at the inlet of each 3WK model is given by

$$\frac{\mathrm{d}P_{\mathrm{in}}}{\mathrm{d}t}+\frac{1}{R_2C}P_{\mathrm{in}}=R_1\frac{\mathrm{d}Q_{\mathrm{in}}}{\mathrm{d}t}+\frac{R_1+R_2}{R_2C}Q_{\mathrm{in}}+\frac{1}{R_2C}P_{\mathrm{out}} \tag{26}$$

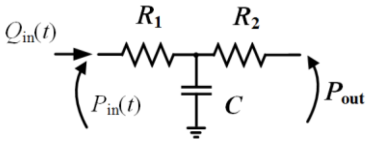

Fig. S2 Three-element Windkessel model (3WK).

The inlet pressure is given by

$$P_{\mathrm{in}}(t)=[P_{\mathrm{in}}(t_0)-R_1Q_{\mathrm{in}}(t_o)-P_{\mathrm{out}}]e^{-\frac{(t-t_0)}{R_2C}}+R_1Q_{\mathrm{in}}(t)+P_{\mathrm{out}}+\frac{e^{-\frac{t}{R_2C}}}{C}\int_{t_0}^{t}e^{\frac{t'}{R_2C}}Q_{\mathrm{in}}(t')\mathrm{d}t'. \tag{27}$$

Assuming periodic flow, Eq. (62) yields the averaged solution

$$R=R_1+R_2=\frac{\bar{P}_{\mathrm{in}}-P_{\mathrm{out}}}{\bar{Q}_{\mathrm{in}}}, \tag{28}$$

where $\bar{P}_{\mathrm{in}}$ is the mean inlet pressure and $\bar{Q}_{\mathrm{in}}$ is the mean inlet flow. Given that P_{out} is known—this was calculated from the exponential decay of pressure during diastole (see Section 1.4)—and R_1 and R_2 are related via Eq. (28), the parameters to be estimated can be reduced from four to two, which are $\widehat{\boldsymbol{\theta}}=\{\hat{R}_2,\hat{C}\}$ and Eq. (6) is given by

$$P_{\mathrm{in}}(t_k)=\left[P_{\mathrm{in}}(t_0)-\left(R-\hat{R}_2\right)Q_{\mathrm{in}}(t_o)-P_{\mathrm{out}}\right]e^{-\frac{(t_k-t_0)}{\hat{R}_2\hat{C}}}+\left(R-\hat{R}_2\right)Q_{\mathrm{in}}(t_k)+P_{\mathrm{out}}+\frac{e^{-\frac{t_k}{\hat{R}_2\hat{C}}}}{\hat{C}}\int_{t_0}^{t_k}e^{\frac{t'}{\hat{R}_2\hat{C}}}Q_{\mathrm{in}}(t')\mathrm{d}t'+e(t_k). \tag{29}$$

The first-order and second-order derivatives of the cost function are given by Eqs. (30)–(39),

$$\frac{\partial f}{\partial\hat{R}_2}=Q_{\mathrm{in}}(t_o)E_1+\left[P_{\mathrm{in}}(t_0)-\left(R-\hat{R}_2\right)Q_{\mathrm{in}}(t_o)-P_{\mathrm{out}}\right]\frac{t_k-t_0}{\hat{R}_2^2\hat{C}}E_1-Q_{\mathrm{in}}(t_k)+\frac{t_k}{\hat{R}_2^2\hat{C}^2}E_2I_1-\frac{1}{\hat{R}_2^2\hat{C}^2}E_2I_2 \tag{30}$$

$$\frac{\partial f}{\partial\hat{C}}=\left[P_{\mathrm{in}}(t_0)-\left(R-\hat{R}_2\right)Q_{\mathrm{in}}(t_o)-P_{\mathrm{out}}\right]\frac{t_k-t_0}{\hat{R}_2\hat{C}^2}E_1+\frac{t_k-\hat{R}_2\hat{C}}{\hat{R}_2\hat{C}^3}E_2I_1-\frac{1}{\hat{R}_2\hat{C}^3}E_2I_2 \tag{31}$$

$$\frac{\partial^2 f}{\partial \hat{R}_2^2} = 2Q_{\mathrm{in}}(t_o)\frac{t_k - t_0}{\hat{R}_2^2 \hat{C}} E_1 + \left[P_{\mathrm{in}}(t_0) - \left(R - \hat{R}_2\right)Q_{\mathrm{in}}(t_o) - P_{\mathrm{out}}\right]\frac{(t_k - t_0)^2 - 2(t_k - t_0)\hat{R}_2\hat{C}}{\hat{R}_2^4 \hat{C}^2} E_1 + \frac{t_k^2 - 2t_k R_2 C}{\hat{R}_2^4 \hat{C}^3} E_2 I_1 - \frac{2t_k + 2\hat{R}_2\hat{C}}{\hat{R}_2^4 \hat{C}^3} E_2 I_2 + \frac{1}{R_2^4 C^3} E_2 I_3 \quad (32)$$

$$\frac{\partial^2 f}{\partial \hat{C}^2} = \left[P_{\mathrm{in}}(t_0) - \left(R - \hat{R}_2\right)Q_{\mathrm{in}}(t_o) - P_{\mathrm{out}}\right]\frac{(t_k - t_0)^2 - 2(t_k - t_0)\hat{R}_2\hat{C}}{\hat{R}_2^4 \hat{C}^4} E_1 + \frac{t_k\left(t_k - \hat{R}_2\hat{C}\right) - 3\left(t_k - \hat{R}_2\hat{C}\right)\hat{R}_2\hat{C} - \hat{R}_2^2\hat{C}^2}{\hat{R}_2^2 \hat{C}^5} E_2 I_1 + \frac{4\hat{R}_2\hat{C} - 2t_k}{\hat{R}_2^2 \hat{C}^5} E_2 I_2 + \frac{1}{\hat{R}_2^2 \hat{C}^5} E_2 I_3 \quad (33)$$

$$\frac{\partial^2 f}{\partial \hat{R}_2 \hat{C}} = \frac{\partial^2 f}{\partial \hat{C} \hat{R}_2} = \frac{t_k - t_0}{\hat{R}_2 \hat{C}} Q_{\mathrm{in}}(t_o) E_1 + \left[P_{\mathrm{in}}(t_0) - \left(R - \hat{R}_2\right) - P_{\mathrm{out}}\right]\frac{(t_k - t_0)^2 - 2(t_k - t_0)\hat{R}_2\hat{C}}{\hat{R}_2^3 \hat{C}^3} E_1 + \frac{t_k(t_k - \hat{R}_2\hat{C}) - t_k\hat{R}_2\hat{C}}{\hat{R}_2^3 \hat{C}^4} E_2 I_1 + \frac{2\hat{R}_2\hat{C} - 2t_k}{\hat{R}_2^3 \hat{C}^4} E_2 I_2 + \frac{1}{\hat{R}_2^3 \hat{C}^4} E_2 I_3, \quad (34)$$

where

$$E_1 = e^{-\frac{(t_k - t_0)}{\hat{R}_2 \hat{C}}} \quad (35)$$

$$E_2 = e^{-\frac{t_k}{\hat{R}_2 \hat{C}}} \quad (36)$$

$$I_1 = \int_{t_0}^{t_k} e^{\frac{t'}{\hat{R}_2 \hat{C}}} Q_{\mathrm{in}}(t')\mathrm{d}t' \quad (37)$$

$$I_2 = \int_{t_0}^{t_k} t' e^{\frac{t'}{\hat{R}_2 \hat{C}}} Q_{\mathrm{in}}(t')\mathrm{d}t' \quad (38)$$

$$I_3 = \int_{t_0}^{t_k} (t')^2 e^{\frac{t'}{\hat{R}_2 \hat{C}}} Q_{\mathrm{in}}(t')\mathrm{d}t' \quad (39)$$

The integrals in Eqs. (37)–(39) were numerically calculated using the trapezoidal method.

This parameter estimation methodology is referred to as PE3; i.e.,

$$\left\{\hat{R}_2, \hat{C}\right\} = \mathrm{PE3}(Q_{\mathrm{in}}(t), P_{\mathrm{in}}(t), P_{\mathrm{out}}). \quad (40)$$

1.6 Parameter estimation for the upper and lower body models (PE4 and PE5)

Upper and lower body models are similar to the vascular bed model depicted in **Fig. S3**. The parameters of this model are the arterial, capillary, and venous resistances, R_a, R_c, and R_v, respectively, and the arterial and venous capacitances C_a and C_v, respectively.

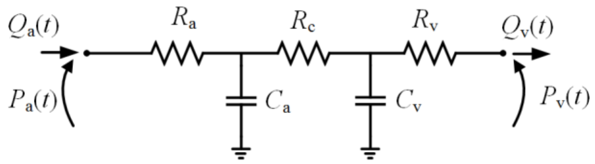

Fig. S3 Vascular bed model.

Assuming that the flow and pressure waveforms at the boundaries $Q_a(t)$, $Q_v(t)$, $P_a(t)$ and $P_v(t)$ are available and the model parameters R_a and C_a are known, then the parameters to be estimated are R_c, R_v, and C_v.

For periodic flow, the mean arterial blood flow, $\bar{Q}_a$, mean arterial blood pressure, $\bar{P}_a$, and mean venous pressure, $\bar{P}_v$, are related through

$$\frac{\bar{P}_a - \bar{P}_v}{\bar{Q}_a} = R_a + R_c + R_v = R. \quad (41)$$

The additional equations of the model include

$$\begin{aligned} P_a(t) = {} & e^{-\frac{(t-t_0)}{R_c C_a}}[P_a(t_o) - R_a Q_a(t_0)] + R_a Q_a(t) \\ & + \frac{e^{-\frac{t}{R_c C_a}}}{C_a}\int_{t_0}^{t} e^{\frac{t'}{R_c C_a}}\left[Q_a(t') + \frac{P_v(t') + (R - R_a - R_c)Q_v(t')}{R_c}\right]\mathrm{d}t' \end{aligned} \quad (42)$$

$$\begin{aligned} P_a(t) = {} & P_a(t_o) + R_a[Q_a(t) - Q_a(t_0)] - \frac{C_v}{C_a}[P_v(t) - P_v(t_0)] - \frac{C_v}{C_a}R_v[Q_v(t) - Q_v(t_0)] \\ & + \int_{t_0}^{t} \frac{1}{C_a}[Q_a(t') - Q_v(t')]\mathrm{d}t' \end{aligned} \quad (43)$$

Two independent optimization processes were carried out in three steps, as described below. First, R_c was estimated from Eq. (42). Then, R_v was calculated from Eq. (41). Finally, C_v was estimated from Eq. (43).

1.6.1 Estimation of capillary resistance

From Eq. (42), the only parameter left to be estimated is $\hat{\boldsymbol{\theta}} = \hat{R}_c$. Therefore, Eq. (6) is given by

$$\begin{aligned} P_a(t_k) = {} & e^{-\frac{(t_k-t_0)}{\hat{R}_c C_a}}[P_a(t_o) - R_a Q_a(t_0)] + R_a Q_a(t_k) \\ & + \frac{e^{-\frac{t_k}{\hat{R}_c C_a}}}{C_a}\int_{t_0}^{t_k} e^{\frac{t'}{\hat{R}_c C_a}}\left[Q_a(t') + \frac{P_v(t') + \left(R - R_a - \hat{R}_c\right)Q_v(t')}{\hat{R}_c}\right]\mathrm{d}t' + e(t_k) \end{aligned} \quad (44)$$

The first-order and second-order derivatives of the cost function are given by Eqs. (45)–(51),

$$\frac{\partial f}{\partial \hat{R}_\mathrm{c}} = [P_\mathrm{a}(t_0) - R_\mathrm{a}Q_\mathrm{a}(t_o)]\frac{t_k - t_0}{\hat{R}_\mathrm{c}^2 C_\mathrm{a}}E_1 + \frac{t_k}{\hat{R}_c^2 C_\mathrm{a}^2}E_2 I_1 - \frac{1}{C_\mathrm{a}}E_2 I_2, \tag{45}$$

$$\frac{\partial^2 f}{\partial \hat{R}_\mathrm{c}^2} = \frac{t_k - t_0 - 2\hat{R}_\mathrm{c}C_\mathrm{a}}{\hat{R}_c^4 C_\mathrm{a}^2}(t_k - t_0)[P_\mathrm{a}(t_0) - R_\mathrm{a}Q_\mathrm{a}(t_o)]E_1 + \frac{(t_k - 2\hat{R}_\mathrm{c}C_\mathrm{a})t_k}{\hat{R}_\mathrm{c}^4 C_\mathrm{a}^3}E_2 I_1 - 2\frac{t_k}{\hat{R}_\mathrm{c}^2 C_\mathrm{a}^2}E_2 I_2 + \frac{1}{C_\mathrm{a}}E_2 I_3, \tag{46}$$

where

$$E_1 = e^{-\frac{(t_k - t_0)}{\hat{R}_\mathrm{c}C_\mathrm{a}}} \tag{47}$$

$$E_2 = e^{-\frac{t_k}{\hat{R}_\mathrm{c}C_\mathrm{a}}} \tag{48}$$

$$I_1 = \int_{t_0}^{t_k} e^{\frac{t'}{\hat{R}_\mathrm{c}C_\mathrm{a}}}\left[Q_\mathrm{a}(t') + \frac{P_\mathrm{v}(t') + \left(R - R_\mathrm{a} - \hat{R}_\mathrm{c}\right)Q_\mathrm{v}(t')}{\hat{R}_\mathrm{c}}\right]\mathrm{d}t' \tag{49}$$

$$I_2 = \int_{t_0}^{t_k} e^{\frac{t'}{\hat{R}_\mathrm{c}C_\mathrm{a}}}\left\{\frac{t'}{\hat{R}_\mathrm{c}^2 C_\mathrm{a}}\left[Q_\mathrm{a}(t') + \frac{P_\mathrm{v}(t') + \left(R - R_\mathrm{a} - \hat{R}_\mathrm{c}\right)Q_\mathrm{v}(t')}{\hat{R}_\mathrm{c}}\right] + \frac{P_\mathrm{v}(t') + (R - R_\mathrm{a})Q_\mathrm{v}(t')}{\hat{R}_\mathrm{c}^2}\right\}\mathrm{d}t' \tag{50}$$

$$\begin{aligned} I_3 = \int_{t_0}^{t_k} e^{\frac{t'}{\hat{R}_\mathrm{c}C_\mathrm{a}}}\Bigg\{&\frac{t'}{\hat{R}_\mathrm{c}^2 C_\mathrm{a}}\Bigg[\frac{t'}{\hat{R}_\mathrm{c}^2 C_\mathrm{a}}\left(Q_\mathrm{a}(t') + \frac{P_\mathrm{v}(t') + \left(R - R_\mathrm{a} - \hat{R}_\mathrm{c}\right)Q_\mathrm{v}(t')}{\hat{R}_\mathrm{c}}\right) \\ &+ \frac{P_\mathrm{v}(t') + (R - R_\mathrm{a})Q_\mathrm{v}(t')}{\hat{R}_c^2}\Bigg] \\ &+ \frac{2t'}{\hat{R}_\mathrm{c}^3 C_\mathrm{a}}\left(Q_\mathrm{a}(t') + \frac{P_\mathrm{v}(t') + \left(R - R_\mathrm{a} - \hat{R}_\mathrm{c}\right)Q_\mathrm{v}(t')}{\hat{R}_\mathrm{c}}\right) \\ &+ \frac{t'}{\hat{R}_\mathrm{c}^4 C_\mathrm{a}}\left(P_\mathrm{v}(t') + (R - R_\mathrm{a})Q_\mathrm{v}(t')\right) + 2\frac{P_\mathrm{v}(t') + (R - R_\mathrm{a})Q_\mathrm{v}(t')}{\hat{R}_\mathrm{c}^3}\Bigg\}\mathrm{d}t' \end{aligned} \tag{51}$$

The integrals in Eqs. (49)–(51) were numerically calculated using the trapezoidal method.

This parameter estimation methodology is referred to as PE4; i.e.,

$$\hat{R}_\mathrm{c} = \mathrm{PE4}(P_\mathrm{a}(t), P_\mathrm{v}(t), Q_\mathrm{a}(t), Q_\mathrm{v}(t), R_\mathrm{a}, C_\mathrm{a}) \tag{52}$$

1.6.2 Estimation of venous capacitance

From Eq. (43), the only parameter left to be estimated is $\widehat{\boldsymbol{\theta}} = \hat{C}_\mathrm{v}$. Therefore, Eq. (6) is given by

$$P_\mathrm{a}(t_k) = P_\mathrm{a}(t_o) + R_\mathrm{a}[Q_\mathrm{a}(t_k) - Q_\mathrm{a}(t_0)] - \frac{\hat{C}_\mathrm{v}}{C_\mathrm{a}}[P_\mathrm{v}(t_k) - P_\mathrm{v}(t_0)] - \frac{\hat{C}_\mathrm{v}}{C_\mathrm{a}}R_\mathrm{v}[Q_\mathrm{v}(t_k) - Q_\mathrm{v}(t_0)] + \int_{t_0}^{t}\frac{1}{C_\mathrm{a}}[Q_\mathrm{a}(t') - Q_\mathrm{v}(t')]\mathrm{d}t' + e(t_k). \tag{53}$$

The first-order and second-order derivatives of the cost function are given by Eqs. (54) and (55),

$$\frac{\partial f}{\partial \hat{C}_\mathrm{v}} = -\frac{1}{C_\mathrm{a}}\left[P_\mathrm{v}(t_k) - P_\mathrm{v}(t_0) + R_\mathrm{v}\big(Q_\mathrm{v}(t_k) - Q_\mathrm{v}(t_0)\big)\right], \tag{54}$$

$$\frac{\partial^2 f}{\partial \hat{C}_\mathrm{v}^2} = 0. \tag{55}$$

This parameter estimation methodology is referred to as PE5; i.e.,

$$\hat{C}_\mathrm{v} = \mathrm{PE5}(P_\mathrm{a}(t), P_\mathrm{v}(t), Q_\mathrm{a}(t), Q_\mathrm{v}(t), R_\mathrm{a}, C_\mathrm{a}, R_\mathrm{v}). \tag{56}$$

Table S1 Parameter values for Submodel 1: 0-D Heart model. BC: Boundary condition.

**INLET BC: PULMONARY ARTERIAL FLOW**	
$Q_{\mathrm{PA}}(t)$ (ml/s)	$Q_{\mathrm{LPA}}(t) + Q_{\mathrm{RPA}}(t)$
**OUTLET BC: 3WK MODEL**	
$R_{1,\mathrm{AAo}}$ (mmHg·s/ml)	0.1476
$R_{2,\mathrm{AAo}}$ (mmHg·s/ml)	0.9536
C_{AAo} (mmHg·s/ml)	3.9067
P_{atrium} (mmHg)	4.2807
**HEART MODEL PARAMETERS**	
Atrium parameters	
$K_{\mathrm{s,A}}$ (s/ml)	$7.5\cdot 10^{-4}$
$E_{\mathrm{min,A}}$ (mmHg/ml)	0.2
$E_{\mathrm{max,A}}$ (mmHg/ml)	0.4
$V_{0,\mathrm{A}}$ (ml)	5.5
$m_{1,\mathrm{A}}$ (–)	20
$m_{2,\mathrm{A}}$ (–)	30
$\tau_{1,\mathrm{A}}/T$ (–)	0.1
$\tau_{2,\mathrm{A}}/T$ (–)	0.25

$t_{offset,A}/T$ (–)	0.7
Atrioventricular valve parameters	
$L_{eff,AVV}$ (cm)	4
$A_{ann,AVV}$ (cm^2)	4.5
$K_{vo,AVV}$ ($mmHg^{-1}\cdot s^{-1}$)	8
$K_{vc,AVV}$ ($mmHg^{-1}\cdot s^{-1}$)	8
Ventricle parameters	
$K_{s,V}$ (s/ml)	$1.4\cdot 10^{-4}$
$E_{min,V}$ (mmHg/ml)	0.065
$E_{max,V}$ (mmHg/ml)	1.97
$V_{0,V}$ (ml)	10.5
$m_{1,V}$ (–)	1.3
$m_{2,V}$ (–)	30
$\tau_{1,V}/T$ (–)	0.18
$\tau_{2,V}/T$ (–)	0.45
$t_{offset,V}/T$ (–)	0
Aortic valve parameters	
$L_{eff,AoV}$ (cm)	8
$A_{ann,AoV}$ (cm^2)	1.68
$K_{vo,AoV}$ ($mmHg^{-1}\cdot s^{-1}$)	4
$K_{vc,AoV}$ ($mmHg^{-1}\cdot s^{-1}$)	2

Table S2 Parameter values for Submodel 2: 1-D/0-D Systemic arterial system model. BC: Boundary condition.

**INLET BC: ASCENDING AORTIC FLOW**	
$Q_{AAo}(t)$ (ml/s)	$Q_{AAo}(t)$
**OUTLET BC: 3WK MODELS**	
P_{out} (mmHg)	36.1691
$R_{1,BCA}$ (mmHg·s/ml)	0.49158
$R_{2,BCA}$ (mmHg·s/ml)	0.27751
C_{BCA} (mmHg·s/ml)	2.1437
$R_{1,LCCA}$ (mmHg·s/ml)	2.1679
$R_{2,LCCA}$ (mmHg·s/ml)	1.2238
C_{LCCA} (mmHg·s/ml)	0.48609
$R_{1,DAo}$ (mmHg·s/ml)	0.2646
$R_{2,DAo}$ (mmHg·s/ml)	0.6204
C_{DAo} (mmHg·s/ml)	2.4921
**1D VESSEL PARAMETERS**	
$c_{d,Ao}$ (m/s)	5.3541

Table S3 Parameter values for Submodel 3: 1-D/0-D TCPC model. BC: Boundary condition.

**INLET BC: SVC AND IVC FLOWS**	
$Q_{SVC}(t)$ (ml/s)	$Q_{SVC}(t)$
$Q_{IVC}(t)$ (ml/s)	$Q_{IVC}(t)$
**OUTLET BC: 3WK MODELS**	

P_{atrium} (mmHg)	4.2807
R_{RPA} (mmHg·s/ml)	0.17199
R_{LPA} (mmHg·s/ml)	0.24735
**1D VESSEL PARAMETERS**	
$c_{\text{d,TCPC}}$ (m/s)	2.8054

Table S4 Parameter values for Submodel 4: 1-D/0-D Coupled aorta–TCPC model. BC: Boundary condition.

**INLET BC: ASCENDING AORTIC FLOWS**	
$Q_{\text{AAo}}(t)$ (ml/s)	$Q_{\text{AAo}}(t)$
**OUTLET BC: 3WK MODELS**	
P_{atrium} (mmHg)	4.2807
R_{RPA} (mmHg·s/ml)	0.17199
R_{LPA} (mmHg·s/ml)	0.24735
**1D VESSEL PARAMETERS**	
$c_{\text{d,Ao}}$ (m/s)	5.3541
$c_{\text{d,TCPC}}$ (m/s)	2.8054
**AORTA–TCPC COUPLING PARAMETERS**	
Upper body	
R_{a1} (mmHg·s/ml)	0.5086
R_{a2} (mmHg·s/ml)	2.2430
R_{c1} (mmHg·s/ml)	1.3305
R_{v1} (mmHg·s/ml)	0.0054

C_{a1} (ml/mmHg)	2.4563
C_{v1} (ml/mmHg)	23.3356
Lower body	
R_{a4} (mmHg·s/ml)	0.2646
R_{c2} (mmHg·s/ml)	0.5767
R_{v2} (mmHg·s/ml)	1.4841
C_{a2} (ml/mmHg)	2.4921
C_{v2} (ml/mmHg)	0.1897

2 Supplementary results

Fig. S4 shows a schematic representation of the model for the specific patient analysed in this study. In the schematic, each 1-D model arterial segment is identified by its domain (circled) and boundary node numbers. For example, the domain number of the ascending aorta (AAo) is 1, and its boundary nodes are 1 and 2. The left pulmonary artery (LPA) was divided into two domains (7 and 8), since its calibre was reduced in the studied patient due to the compression force exerted by the aorta. **Table S1** to **Table S4** show the model parameters that were estimated for each submodel. **Fig. S5** compares time-varying haemodynamic quantities measured *in vivo* and computed by the complete model and each submodel. The relative errors of these quantities with respect to their corresponding *in vivo* measurements calculated using Eqs. (31) and (32) in the main manuscript are presented in **Table S5**.

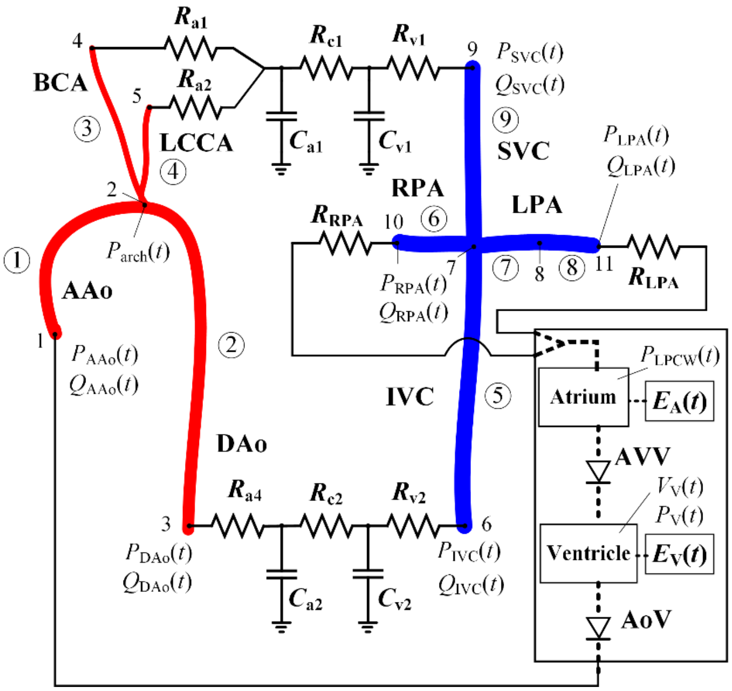

Fig. S4 Schematic representation of the patient-specific model used in this study. Circled numbers indicate 1-D domains and numbers indicate node numbers. The locations of the *in vivo* pressure (*P*), flow (*Q*), and volume (*V*) time-varying measurements shown in **Fig. 1** are also indicated.

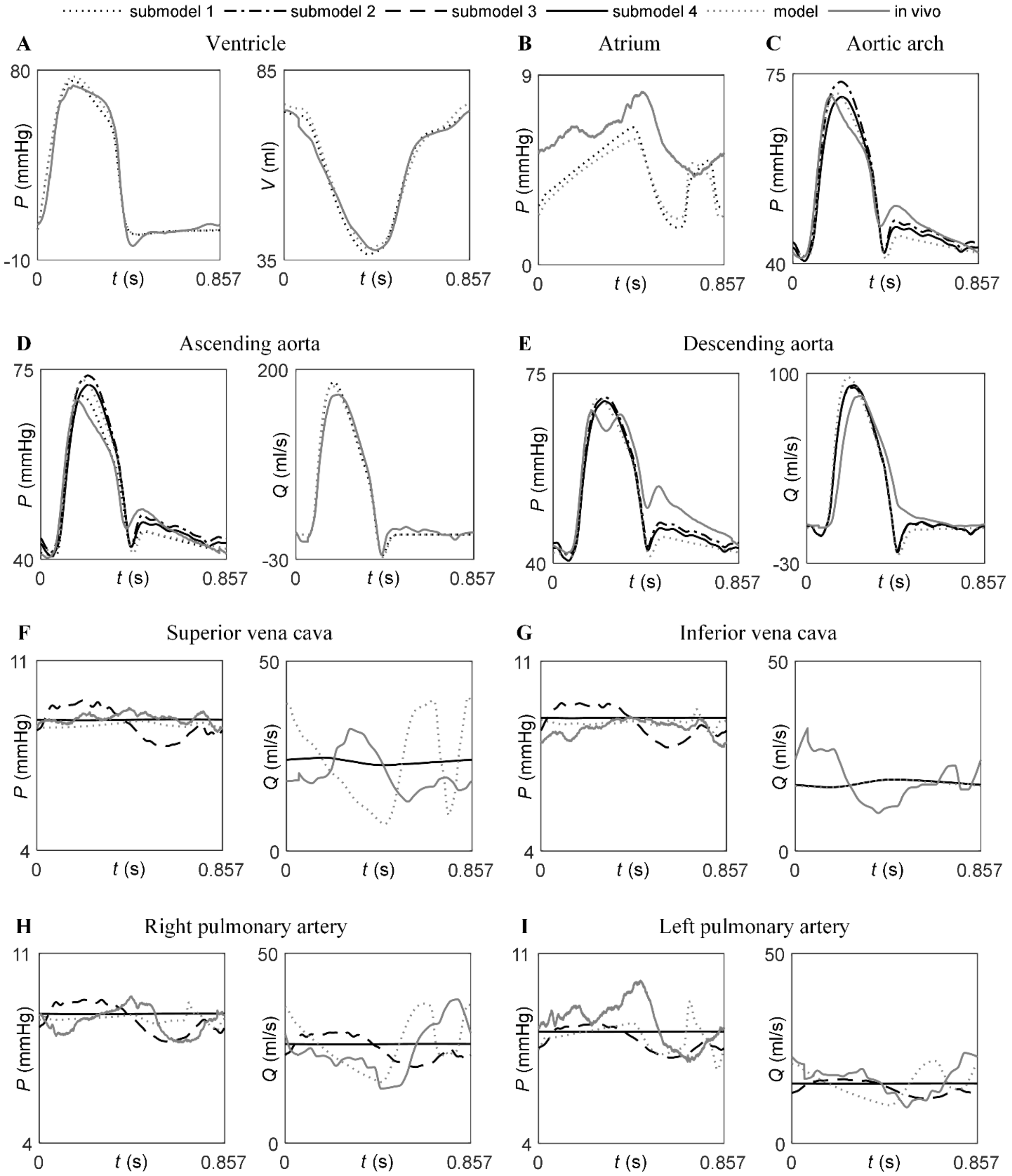

Fig. S5 Simulated vs. *in vivo* hemodynamic quantities at baseline, for all models. Comparison between time-varying hemodynamics quantities measured *in vivo* and computed by the closed-loop model and submodels shown in **Figs. 2** and **3**, respectively, of the main manuscript. Time-varying pressure (P) and volume (V) in the ventricle (***A***), P in the atrium (***B***) and aortic arch (***C***), and P and blood flow (Q) waveforms at ascending aorta (***D***), descending aorta (***E***), SVC (***F***), IVC (***G***), RPA (***H***), and LPA (***I***),simulated by Submodel 1 (dotted lines), Submodel 2 (dashed-dotted lines), Submodel 3 (dashed lines), Submodel 4 (black solid lines), closed-loop model (thin dotted lines), and measured *in vivo* (grey solid lines). All six data types are not available for all panels.

Table S5 Comparison of relative errors in the mean value (in pressure, $\varepsilon_{\bar{P}}$, and in flow rate, $\varepsilon_{\bar{Q}}$) and mean relative point-to-point errors (ε_P and ε_Q) among the four submodels (SMs) and in the closed-loop model (M). The absolute value of the mean errors is used to calculate the Average row. AAo: ascending aorta; Arch: aortic arch; DAo: descending aorta; IVC: inferior vena cava; SVC: superior vena cava; RPA: right pulmonary artery; LPA: left pulmonary artery; SM1: Submodel 1; SM2: Submodel 2; SM3: Submodel 3; SM4: Submodel 4. Zero error means that this quantity was prescribed; NA: not available because no measurement was available.

**Location**	$\varepsilon_{\bar{P}}$ **(%)**					ε_P **(%)**					$\varepsilon_{\bar{Q}}$ **or** $\varepsilon_{\bar{V}}$ **(%)**					ε_Q **or** ε_V **(%)**				
	**SM1**	**SM2**	**SM3**	**SM4**	**M**	**SM1**	**SM2**	**SM3**	**SM4**	**M**	**SM1**	**SM2**	**SM3**	**SM4**	**M**	**SM1**	**SM2**	**SM3**	**SM4**	**M**
AAo	-1.64	3.38		1.27	0.51	3.46	4.95		4.42	4.72	-0.36	0		0	-0.15	3.97	0		0	3.75
Arch		0.55		-1.95	-2.72		4.02		4.44	4.91		NA		NA	NA		NA		NA	NA
DAo		-3.39		-5.03	-5.81		5.39		6.59	7.53		-2.40		-1.54	-1.68		10.75		10.29	13.24
IVC			2.45	4.31	2.23			7.14	4.44	3.05			0	-2.99	-3.30			0	16.11	16.29
SVC			-2.05	-0.62	-2.77			6.14	1.65	2.85			0	9.29	9.10			0	17.99	41.26
RPA			1.04	3.30	1.12			6.18	5.41	4.97			1.36	4.31	4.12			18.89	15.03	15.50
LPA			-7.49	-3.95	-6.26			7.78	8.57	9.62			-9.70	-4.65	-4.99			12.25	14.22	18.88
Ventricle	0.41				1.86	3.93				3.71	0.14				1.76	2.49				2.86
Average	1.02	2.44	3.26	2.92	2.91	3.69	4.79	6.81	5.07	5.17	0.25	2.40	5.53	4.55	3.59	3.23	10.75	15.57	14.73	15.97

3 References

[1] J. Alastruey, Numerical assessment of time-domain methods for the estimation of local arterial pulse wave speed, Journal of Biomechanics 44 (2011) 885–891. https://doi.org/10.1016/j.jbiomech.2010.12.002.

[2] J. Alastruey, N. Xiao, H. Fok, T. Schaeffter, C.A. Figueroa, On the impact of modelling assumptions in multi-scale, subject-specific models of aortic haemodynamics, Journal of The Royal Society Interface 13 (2016) 20160073. https://doi.org/10.1098/rsif.2016.0073.

[3] J.E. Davies, Z.I. Whinnett, D.P. Francis, K. Willson, R.A. Foale, I.S. Malik, A.D. Hughes, K.H. Parker, J. Mayet, Use of simultaneous pressure and velocity measurements to estimate arterial wave speed at a single site in humans, American Journal of Physiology-Heart and Circulatory Physiology 290 (2006) H878–H885. https://doi.org/10.1152/ajpheart.00751.2005.
